# EFEMP1 promotes the migration and invasion of osteosarcoma via MMP-2 with induction by AEG-1 via NF-κB signaling pathway

**DOI:** 10.18632/oncotarget.3691

**Published:** 2015-03-29

**Authors:** Zhuo Wang, Chuang-Jie Cao, Lei-Lei Huang, Zun-Fu Ke, Can-Jiao Luo, Zhong-Wei Lin, Fen Wang, Yuan-Qi Zhang, Lian-Tang Wang

**Affiliations:** ^1^ Department of Pathology, The First Affiliated Hospital of Sun Yat-sen University, Guangzhou, China; ^2^ Department of Cardiology, The First Affiliated Hospital of Guangdong Pharmaceutical University, Guangzhou, China; ^3^ Department of Vascular Surgery, Affiliated Hospital of Guangdong Medical College, Zhanjiang, China

**Keywords:** osteosarcoma, AEG-1, EFEMP1, MMP-2, NF-κB

## Abstract

The role of epidermal growth factor-containing ﬁbulin-like extracellular matrix protein 1 (EFEMP1) in osteosarcoma remains unknown. Then applying EFEMP1 siRNA, plasmids transfection and adding purified EFEMP1 protein in human osteosarcoma cell lines, and using immunohistochemistry on 113 osteosarcoma tissues, demonstrated that EFEMP1 was a poor prognostic indicator of osteosarcoma; EFEMP1 was specifically upregulated in osteosarcoma and associated with invasion and metastasis *in vitro* and *in vivo*. At the same time, we found a direct regulatory effect of EFEMP1 on MMP-2. Moreover, we firstly found the marked induction of EFEMP1 by oncogenic AEG-1. And EFEMP1 expression was inhibited by the selective inhibitor of NF-κB (PDTC) in osteosarcoma cells. Then we thought that NF-κB pathways might be one of the effective ways which EFEMP1 was induced by AEG-1. Thus, we suggested that EFEMP1 played a part as the mediator between AEG-1 and MMP-2. And NF-κB signaling pathway played an important role in this process. In summary, EFEMP1 was associated with invasion, metastasis and poor prognosis of osteosarcoma patients. EFEMP1 might indirectly enhance the expression of MMP-2, providing a potential explanation for the role of AEG-1 in metastasis. NF-κB pathways might be one of the effective ways which EFEMP1 was induced by AEG-1.

## INTRODUCTION

Osteosarcoma is the most common primary malignant tumor of the bone in children and adolescents with a poor prognosis. However, the etiology and progression events intrinsic to the malignant properties of osteosarcoma remain obscure.

Epidermal growth factor-containing ﬁbulin-like extracellular matrix protein 1 (EFEMP1), also called ﬁbulin-3, is a member of the ﬁbulin family of extracellular glycoproteins which distributed in various human tissues [1]. They modulate the cell morphology, growth, adhesion, and motility, etc [1]. EFEMP1 was initially identiﬁed as a gene with increased expression in senescent and Werner syndrome ﬁbroblasts [2]. Concurrently, EFEMP1 was associated with inherited forms of macular degeneration [3].

The complex functions of ﬁbulins are also evident in the processes associated with tumors. However, little information is available about EFEMP1. The results supporting whether EFEMP1 promotes or inhibits cancer development are conflicting. To date, both tumor suppressive functions and oncogenic activities have been associated with EFEMP1 [4], such as glioma [5, 6]. Furthermore, it was found that EFEMP1 is a binding partner of tissue inhibitor of metalloproteinase 3(TIMP-3). TIMP-3 encodes a member of TIMP family that was originally identified as an inhibitor of matrix metalloproteinase(MMP) [7]. Some researchers presumed that EFEMP1 regulated matrix composition and negatively affects tumor growth, invasion and angiogenesis by interacting with TIMP-3 and downregulating MMPs.

In contrast, there is little evidence for the pro-tumor effects of EFEMP1. For instance, the overexpression of EFEMP1 contributes to the enhancement of tumor growth in pancreatic carcinoma cells by binding the EGF receptor and activating the MAPK and Akt pathways [8]. In addition, plasma fibulin-3(EFEMP1) levels distinguish healthy persons who have been exposed to asbestos from patients with mesothelioma [9]. Due to the controversial role of EFEMP1 in tumor development and the lack of osteosarcoma research, we aimed to evaluate the potential function of EFEMP1 in osteosarcoma.

AEG-1, also known as metadherin(MTDH) or lyric, plays a functional role in all important hallmarks of an aggressive tumor, including transformation, invasion, metastasis, angiogenesis, evasion of apoptosis and chemoresistance [10]. Therefore, AEG-1 has emerged as an important oncogene in most cancers, such as glioma, neuroblastoma and renal cancer [11-13].

AEG-1 increases the invasiveness of HeLa cells by activating the NF-κB pathway [14]. AEG-1 expression is significantly induced by oncogenic Ha-ras through the PI3K/AKT/GSK3β/c-Myc signaling pathway [15]. In addition, there is an interesting connection between AEG-1 and MMPs. Some results suggest that downregulation of AEG-1 significantly inhibits the expression of MMP-2, 7 and 9 in glioma and lung cancer. Our previous study was the first to show that AEG-1 was highly expressed in osteosarcoma, associated with tumor cell proliferation and invasion. Moreover, AEG-1 plays a crucial role in osteosarcoma progression through MMP-2 [16]. Using an Axon GenePix 4000B microarray scanner (Molecular Devices Corporation), we assayed differentially expressed genes in the osteosarcoma cell line MG63 following transfection with pcDNA3.1-AEG-1. Upregulated genes included those associated with oncogenic genes and those that play the roles in cell invasion, cell growth, evasion of apoptosis, and cell differentiation (data not published). EFEMP1 is one of the genes which are significantly upregulated in MG63 cell transfected with pcDNA3.1-AEG-1. Therefore, one aim of the present study was to confirm that EFEMP1 is induced by AEG-1, and determine whether there is a relationship between EFEMP1 and AEG-1.

Here we found that EFEMP1 is a poor prognostic indicator of osteosarcoma; EFEMP1 was specifically upregulated in osteosarcoma and associated with invasion and metastasis. EFEMP1 might indirectly induce MMP-2 and provide a potential explanation for the role of AEG-1 in metastasis. Moreover, we thought that NF-κB pathways might be one of the effective ways which EFEMP1 was induced by AEG-1. Thus, we suggest that EFEMP1 acted a part as the mediator between AEG-1 and MMP-2.

## RESULTS

### Overexpression of EFEMP1 in osteosarcoma patients

To further investigate whether EFEMP1 was overexpressing in clinical samples of osteosarcoma, immunohistochemistry, western blotting and qRT-PCR analyses were used to evaluate the expression of EFEMP1 at both protein and mRNA levels.

**Figure 1:**
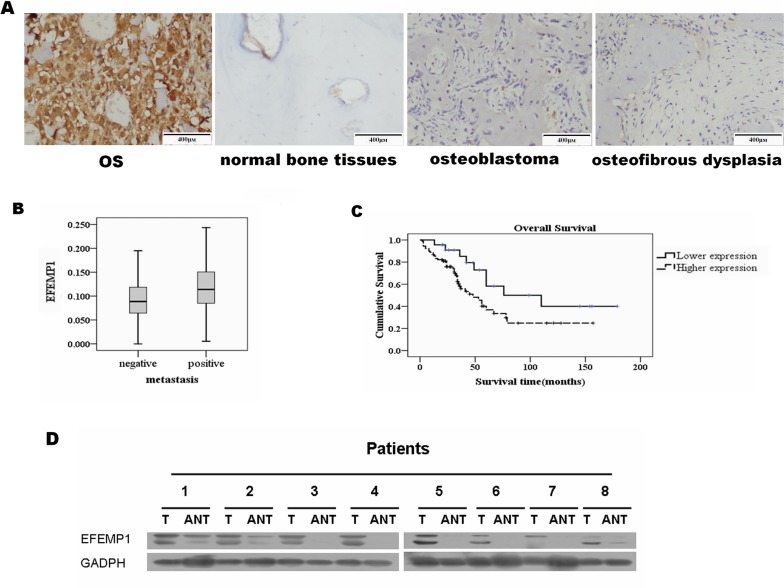
Overexpression of EFEMP1 in osteosarcoma patients (**A**) Immunohistochemistry of EFEMP1 in tissue specimens: EFEMP1 protein was upregulated in osteosarcoma sections compared with normal bone tissue, benign bone tumors and tumor-like bone lesions, as examined by immunohistochemical staining. EFEMP1 was specifically upregulated in osteosarcoma. (**B**) Expression levels of EFEMP1 in the group with hematogenous metastasis versus the group without; p = 0.011. (**C**) Kaplan–Meier curves showed overall survival according to EFEMP1 expression. High EFEMP1 expression (dotted line) was associated with poorer outcomes compared with low EFEMP1 expression (solid line). (**D**) Comparative quantification of EFEMP1 protein in paired primary osteosarcoma tissues (T) and adjacent non-tumor tissues (ANT), with each pair obtained from the same patient. Protein expression levels were normalized to GADPH.

Tumor samples from 113 osteosarcoma patients were collected for immunohistochemistry using anti-EFEMP1 antibodies. EFEMP1 positive signals were located in the cytoplasm of cells by immunohistochemistry. The mean density of EFEMP1 expression in osteosarcoma samples was 0.10342 ± 0.051114. The highest expression level of EFEMP1 was assumed to be a mean EFEMP1 expression density ≧0.06. A mean expression density <0.06 indicated a low level of EFEMP1 expression. Thus, a high expression level of EFEMP1 was detected in 113 osteosarcoma cases (79.6%) using immunohistochemistry. A low level of EFEMP1 expression was found in the remaining osteosarcoma cases, 20 cases of normal bone tissues, 10 cases of osteoblastoma, 10 cases of osteoma, 6 cases of osteoid osteoma and 12 cases of tumor-like bone lesions (osteofibrous dysplasia and fibrous dysplasia; Figure 1A).

113 osteosarcoma patients, whose characteristics are listed in Table 1, were successfully followed up. The correlation between clinicopathological parameters and EFEMP1 expression was confirmed using a Spearman's rank correlation analysis (Table 1). Among these 113 patients, there was a statistically significant difference in Enneking staging system and EFEMP1 expression (*P* = 0.036). Further analysis showed that the association between EFEMP1 and hematogenous metastasis was signiﬁcantly stronger in osteosarcoma patients (r = 0.239, *P* = 0.011) (Figure 1B).

**Table 1:** Correlation between clinicopathological parameters and EFEMP1 expression

Factor	No. 113	EFEMP1 protein expression		*P*
		Low, n(%)	High, n(%)	
Age				0.318
<20	84	0 (0)	84 (100%)	
≥20	29	23 (79.3%)	6 (20.7%)	
Gender				0.662
Male	71	16 (22.5%)	55 (77.5%)	
Female	42	7 (16.7%)	35 (83.3%)	
Tumor size				0.109
<5cm	19	3 (15.8%)	16 (84.2%)	
≥5cm	94	20 (21.3%)	74 (78.7%)	
Tumor position				0.522
Femur	71	14 (19.7%)	57 (80.3%)	
Tibia	27	4(14.8%)	23(85.2%)	
Humerus	6	1(16.7%)	5(83.3%)	
Fibulars	3	0	3(100%)	
Radius	1	0	1(100%)	
irregular bone	5	1(20%)	4(80%)	
Metastasis				0.011*
Absent	59	14 (23.7%)	45 (76.3%)	
Present	54	9 (16.7%)	45 (83.3%)	
Enneking staging				0.036*
II A	8	4(50%)	4(50%)	
II B	51	9(17.6%)	42(82.4%)	
III A	7	2(28.6%)	5(71.4%)	
III B	47	5(10.6%)	42(89.4%)	
Histologic type				0.098
Conventional OS (Osteoblastic)	77	13(16.9%)	64(83.1%)	
Conventional OS (Chondroblastic)	10	4(40%)	6(60%)	
Conventional OS (Fibroblastic)	10	1(10%)	9(90%)	
Telangiectatic OS	9	0	9(100%)	
Parosteal OS	5	2(40%)	3(60%)	
Small cell OS	2	0	2(100%)	

OS: osteosarcoma;

* *P* < 0.05

A multivariate survival analysis indicated that the EFEMP1 expression level was an independent prognostic factor of patient survival (Table 2). Kaplan-Meier survival curves (Figure 1C) showed that the survival rate of patients with a high level of EFEMP1 expression was significantly reduced compared with patients with a low level of EFEMP1 expression (correlation coefficient = −0.198, *P* = 0.036). The log-rank test demonstrated that the survival time between the low and high EFEMP1 expression groups was significantly different (F = 4.182, *P* = 0.041). The average survival time was only 67.517 months in the high EFEMP1 expression group (95% confidence interval, 51.208–83.825 months), whereas it was 106.983 months in the low EFEMP1 expression group (95% confidence interval, 74.959–139.006 months). Taken together, our results suggested that overexpression of EFEMP1 significantly correlated with hematogenous metastasis and poor outcome in osteosarcoma patients.

**Table 2:** Multivariate analyses of various potential prognostic factors in osteosarcoma patients

Factors	RR	95%CI	P
Age	1.034	0.504-2.121	0.927
Gender	1.157	0.615-2.175	0.652
Tumor size	0.543	0.207-1.427	0.215
Tumor position	0.803	0.496-1.300	0.372
Histology	0.917	0.410-2.052	0.833
Metastasis	3.356	1.733-6.497	<0.001**
Enneking staging	0.225	0.149-1.737	0.045*
EFEMP1 expression	6.83	1.50-14.86	<0.001**

RR: relative ratio; CI: confidence interval;

* *P*< 0.05,

** *P*< 0.01.

Furthermore, a comparative analysis of osteosarcoma tumor tissue and paired adjacent non-tumor tissues (ANT) revealed that the mRNA and protein levels of EFEMP1 was elevated in tumor tissues compared with ANT tissues in eight osteosarcoma cases (Figure 1D & Figure S1).

### EFEMP1 knockdown inhibited cell migration, invasion and colony formation in osteosarcoma cells

**Figure 2:**
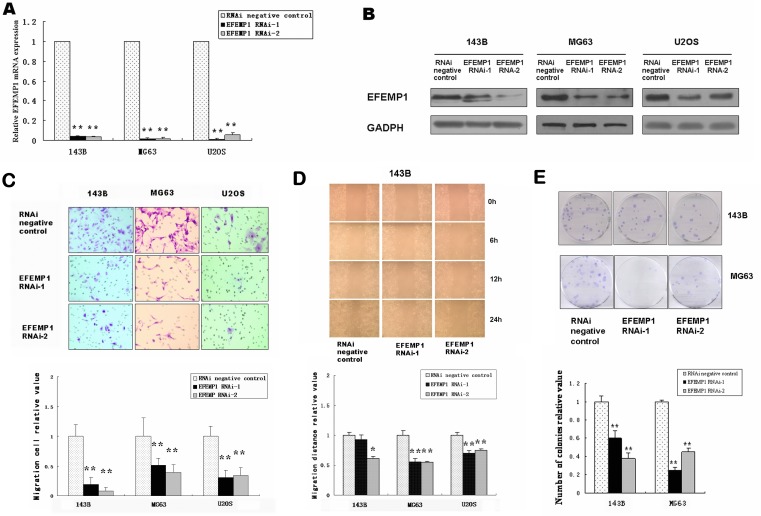
Downregulation of EFEMP1 in osteosarcoma cell lines suppressed migration, invasion and colony formation *in vitro* Small interfering RNA approaches to inhibit the endogenous expression of EFEMP1. (**A**) Quantification of EFEMP1 mRNA levels in EFEMP1 RNAi–transduced osteosarcoma cells. The mRNA expression levels are presented as the increasing fold compared with the negative control cells and were normalized to GAPDH. (**B**) Expression of EFEMP1 following application of siRNA approaches to inhibit the endogenous expression of EFEMP1 in osteosarcoma cell lines 143B, MG63 and U2OS, analyzed by western blot using an anti–EFEMP1 antibody. Protein expression levels were normalized to GADPH. (**C**) Reduced EFEMP1 expression inhibited the invasive ability of osteosarcoma cells. Representative pictures of penetrated cells in lower chamber (upper) and quantification of the indicated cells (lower) using the transwell matrix penetration assay. The 143B (2× 10^4^), MG63 (2× 10^4^) and U2OS (2× 10^4^) were added to the upper chamber in serum-free medium. Migrating cells were scored using a microscope at 100× magnification. Relative quantification of penetrated cells in lower chamber represents the mean of three different experiments. (**D**) Wound-healing assays of 143B osteosarcoma cell revealed that downregulation of EFEMP1 significantly inhibited cell migration compared with the control group. The relative migration distance at 24 hour of osteosarcoma cells were indicated in the bar chart. (**E**) Compared with the control groups, the number of colonies was significantly reduced in the EFEMP1 RNAi group. Each experiment was performed three times in triplicate. * *P* < 0.05, ** *P* < 0.01.

To conﬁrm the effect of EFEMP1 on osteosarcoma cell migration and invasion, siRNA techniques were used to inhibit the endogenous expression of EFEMP1 (Figure 2A and 2B). The different groups of osteosarcoma cells were subjected to matrigel invasion assays and wound-healing assays *in vitro*. The migratory ability of the EFEMP1 siRNA group was approximately 2 to 12 fold lower than that of the negative controls in matrigel invasion assays (Figure 2C). The wound-healing assays *in vitro* indicated that downregulation of EFEMP1 significantly inhibited cell migration compared with the control group (Figure 2D).

The proliferation rate, as determined using the CCK8 assay, did not differ signiﬁcantly between the negative control and cells with downregulated EFEMP1 expression during a 48-h period (Figure S2). In addition, cell apoptosis did not differ between the cells with EFEMP1 siRNA and negative control, as assessed by flow cytometry.

However, colony-forming ability was affected by EFEMP1 siRNA, as evidenced by an approximately 11~75% decrease in colony formation compared with control cells (Figure 2E).

### EFEMP1 expression promoted cell migration, invasion and colony formation in osteosarcoma cells

**Figure 3:**
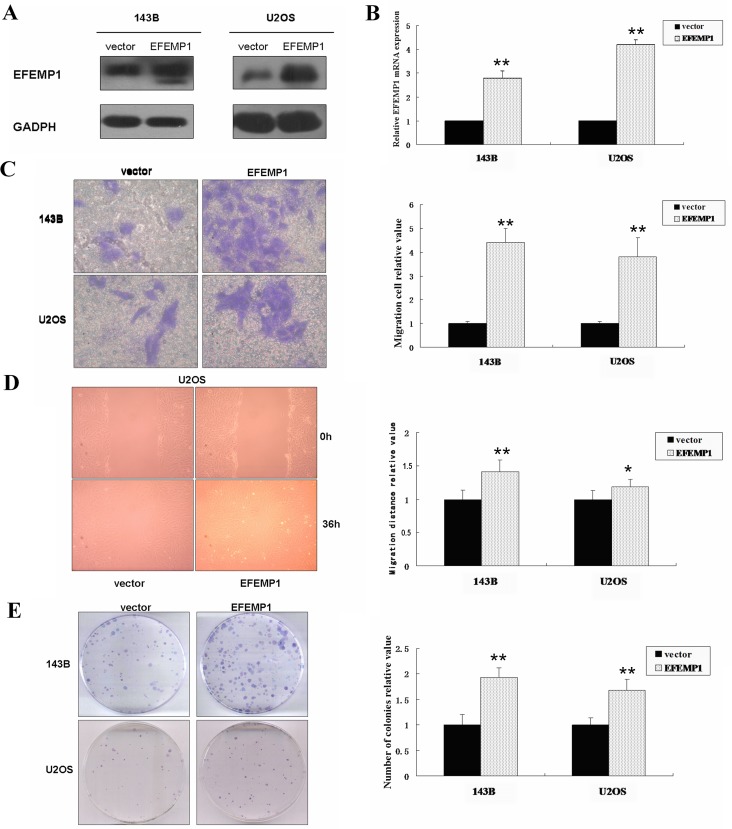
EFEMP1 expression promoted cell migration, invasion and colony formation in osteosarcoma cells *in vitro* (**A**) Expression of EFEMP1 in osteosarcoma cell lines 143B and U2OS stably transfected with EFEMP1 expression plasmid, analyzed by western blot using an anti–EFEMP1 antibody. Protein expression levels were normalized to GADPH. (**B**) Quantification of EFEMP1 mRNA levels in osteosarcoma cell lines 143B and U2OS stably transfected with EFEMP1 expression plasmid. The mRNA expression levels are presented as the increasing fold compared with the empty vector control cells and were normalized to GAPDH. (**C**) Over-expression EFEMP1 expression significantly promoted the invasive ability of osteosarcoma cells. Representative pictures of penetrated cells in lower chamber (left) and quantification of the indicated cells (right) using the transwell matrix penetration assay. The 143B (0.5× 10^4^) and U2OS (2× 10^4^) were added to the upper chamber in serum-free medium. Migrating cells were scored using a microscope at 100× magnification. Relative quantification of penetrated cells in lower chamber represents the mean of three different experiments. (**D**) Wound-healing assays of osteosarcoma cells revealed that over-expression EFEMP1 expression significantly promoted cell migration compared with the empty vector control group. The relative migration distance was indicated in the bar chart. (**E**) Compared with the empty vector control groups, the number of colonies was significantly increased in the over-expression EFEMP1 expression group. Each experiment was performed three times in triplicate. * *P* < 0.05, ** *P* < 0.01.

As shown in Figure 3A and 3B, EFEMP1 mRNA and protein level in 143B and U2OS stably transfected with EFEMP1 expression plasmid was significantly increased about 3-4 fold of the empty vector control groups using real-time PCR and western blot analysis. Matrigel invasion assays showed that the mean number of invasive cells per field of view was significantly more in 143B and U2OS stably transfected with EFEMP1 expression plasmid than that in empty vector control groups (*p*< 0.05; Figure 3C). Because EFEMP1 is an extracellular matrix protein, osteosarcoma cell were treated with purified EFEMP1 protein to induce exogenous overexpression of EFEMP1 in tumor microenvironment. The migratory ability of osteosarcoma cells after treatment with EFEMP1 protein (25, 50, 100, and 200 ng/mL) was higher than that of the negative controls (Figure S3A and S3B).

The wound-healing assays *in vitro* indicated that cell migration was dramatically promoted in 143B and U2OS stably transfected with EFEMP1 expression plasmid than that in empty vector control groups (*p* < 0.01; Figure 3D). Concomitantly, the wound-healing assays indicated that the treatment with EFEMP1 protein (25, 50, 100, and 200 ng/mL) had the similar positive effect (Figure S3C).

Colony formation assays indicated that colony-forming ability was dramatically promoted in 143B and U2OS stably transfected with EFEMP1 expression plasmid than that in empty vector control groups (*P* < 0.01; Figure 3E).

Taken together, our results suggested that EFEMP1 was an important factor in promoting the migration and invasion of osteosarcoma cell lines *in vitro*.

### EFEMP1 expression promoted the tumorigenicity and metastasis of osteosarcoma cells *in vivo*

**Figure 4:**
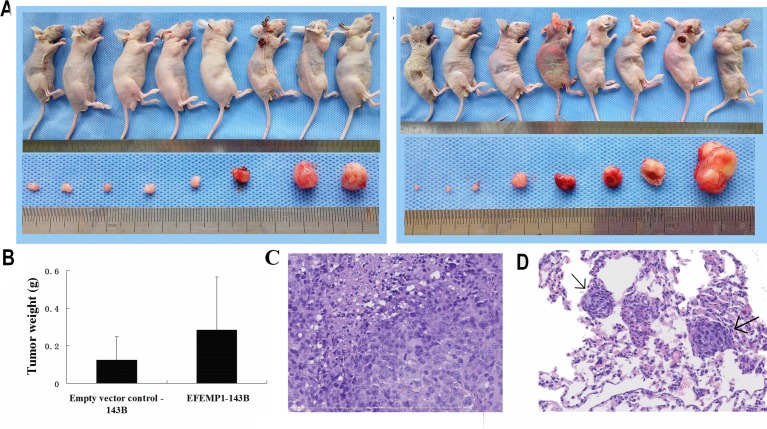
Tumorigenicity of osteosarcoma cells that overexpress EFEMP1 (**A**) Tumorigenicity of the indicated stable transfectants that were injected with empty vector-143B (Left) or EFEMP1-143B cells(Right) when grown for 30 days in the nude mouse model (n = 8 mice per group). (**B**) The average weight of tumors derived from EFEMP1-143B cells group was only slight larger than that of empty vector control group. (**C**) H&E staining were performed on subcutaneous transplantation tumor on EFEMP1-143B cells group. Histologic examination shows the tumor was composed of highy pleomorphic cells. Tumor cells are large and spindle with central, oval nuclei and prominent nucleoli. It is easy to find the necrosis and mitotic. (**D**) H&E staining were performed on the lesions were the osteosarcoma metastasis tumor in lungs of EFEMP1-143B cells group (marking with the arrow). The morphology of metastatic tumor cells are similar with subcutaneous transplantation tumor cells.

To determine whether EFEMP1 affects the tumorigenicity of osteosarcoma cells *in vivo*, EFEMP1-143B or empty vector-143B cells were subcutaneously injected into 8 nude mice and tumor formation was followed for 5 weeks. As shown in Figure 4A and 4B, there was not signiﬁcantly difference between EFEMP1-143B and empty vector-143B cells implanted subcutaneously in BALB/c-nu mice, although the average volume and weight of tumors derived from EFEMP1-143B cells group was only slight larger than that of empty vector control group (*p* > 0.05). However, the number of mice injected with EFEMP1-143B cells with lung metastatic nodules was significantly higher than the mice injected with empty vector-143B (4/8 versus 0/8; *P* < 0.001) through macrography and histological examination. Histological studies confirmed that the lesions were the osteosarcoma metastasis tumor in lungs (Figure 4C and 4D).

**Figure 5:**
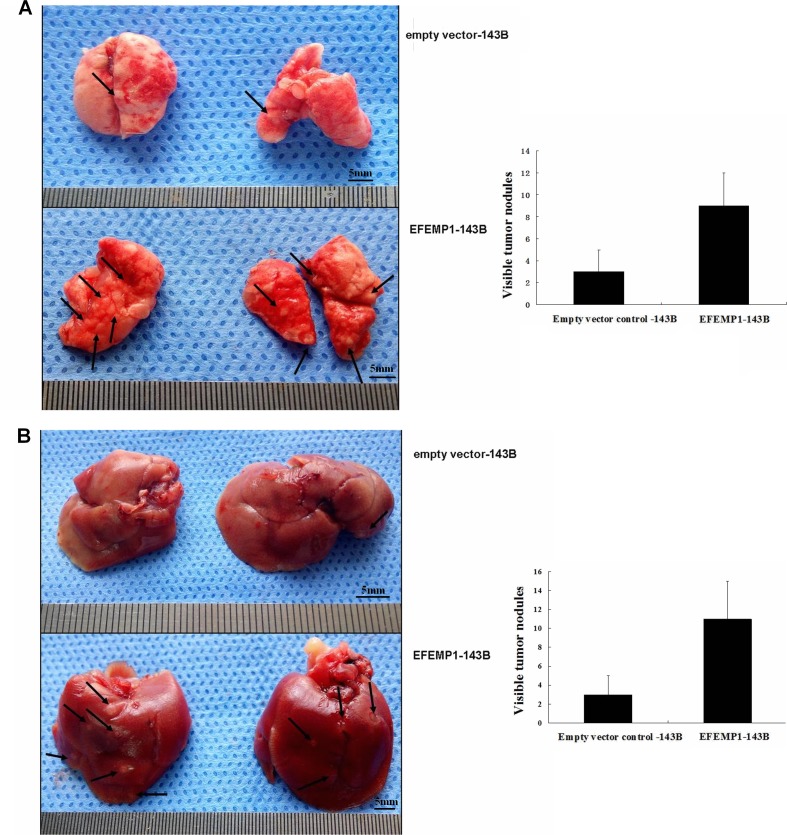
EFEMP1 promotes tumor metastasis *in vivo* through experimental metastasis assay (**A**) Metastatic nodules (arrows) on the surface of the lung(left). The number of visible tumor nodules were quantified on lungs of mice (right). (**B**) Metastatic nodules (arrows) on the surface of the liver (left). The number of visible tumor nodules were quantified on liver of mice (right).

To investigate the effects of EFEMP1 overexpression on metastasis *in vivo*, the experimental metastasis assay was used to compare the metastatic nodules formed in the lungs and livers of BALB/c-nu mice after the inoculation with EFEMP1-143B or empty vector-143B cells. At 4 weeks after injection, the mice were euthanized, and the lungs and livers were harvested. The number of metastatic nodules on the surface of the lungs was significantly lower in mice injected with empty vector-143B cells than in mice injected with EFEMP1-143B cells (3 ± 2 versus 9 ± 3; *P* < 0.001, independent Student's *t* test; Figure 5A). Similarly, the number of metastatic nodules on the surface of the liver was significantly lower in mice injected with empty vector-143B cells than in mice injected with EFEMP1-143B cells (3 ± 2 versus 11 ± 4; *P* < 0.001, independent Student's *t* test; Figure 5B). Histological studies confirmed that the lesions were caused by extravasation and subsequent tumor growth of osteosarcoma cells into the lungs and livers.

### EFEMP1 expression increased the expression and activity of MMP2 without affecting TIMP-3 in osteosarcoma cells

**Figure 6:**
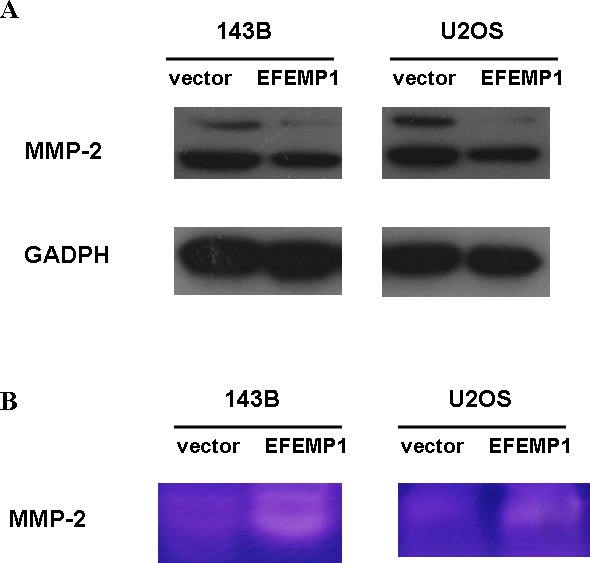
EFEMP1-mediated tumor cell migration and invasion was linked to MMP-2 (**A**) MMP-2 protein levels increased in 143B and U2OS cells stably transfected with EFEMP1 expression plasmid compared with the empty vector control groups, as assessed by western blot analysis. (**B**) Conditioned medium harvested from treated osteosarcoma cells was analyzed by gelatin zymography. White bands represent MMP-2-mediated gelatin digestion. The results demonstrated that MMP-2 activity was increased in 143B and U2OS cells stably transfected with EFEMP1 expression plasmid compared with the empty vector control groups.

To demonstrate the association between EFEMP1-mediated tumor cell invasion and MMP-2, we showed that MMP-2 protein and mRNA levels increased in 143B and U2OS cells stably transfected with EFEMP1 expression plasmid compared with the empty vector control groups (Figure 6A and Figure S4). Moreover, gelatin zymography was conducted to examine the activity of MMP-2 in the conditioned medium of treated osteosarcoma cells. The activity of MMP-2 was increased in 143B and U2OS cells stably transfected with EFEMP1 expression plasmid compared with the empty vector control groups (Figure 6B).

This finding was further confirmed in MMP-2 protein and mRNA levels decreased after the application of EFEMP1 siRNA in osteosarcoma cells compared with the negative controls (Figure S5A, S5B and S5C). Moreover, the activity of MMP-2 was abolished in osteosarcoma cells with suppressed EFEMP1 expression (Figure S5D).

These results suggested that there was a direct regulatory effect of EFEMP1 on MMP-2 expression and activity in osteosarcoma cells.

Because some researches have demonstrated a relationship between EFEMP1 and TIMP-3, we investigated the association between EFEMP1 and TIMP-3. Unfortunately, the results did not support the correlation between EFEMP1 and TIMP-3 with respect to protein and RNA levels (Figure S6).

### EFEMP1 is a downstream target molecule of astrocyte elevated gene-1 (AEG-1), mediating its tumor-migrating and invasive effects in osteosarcoma

**Figure 7:**
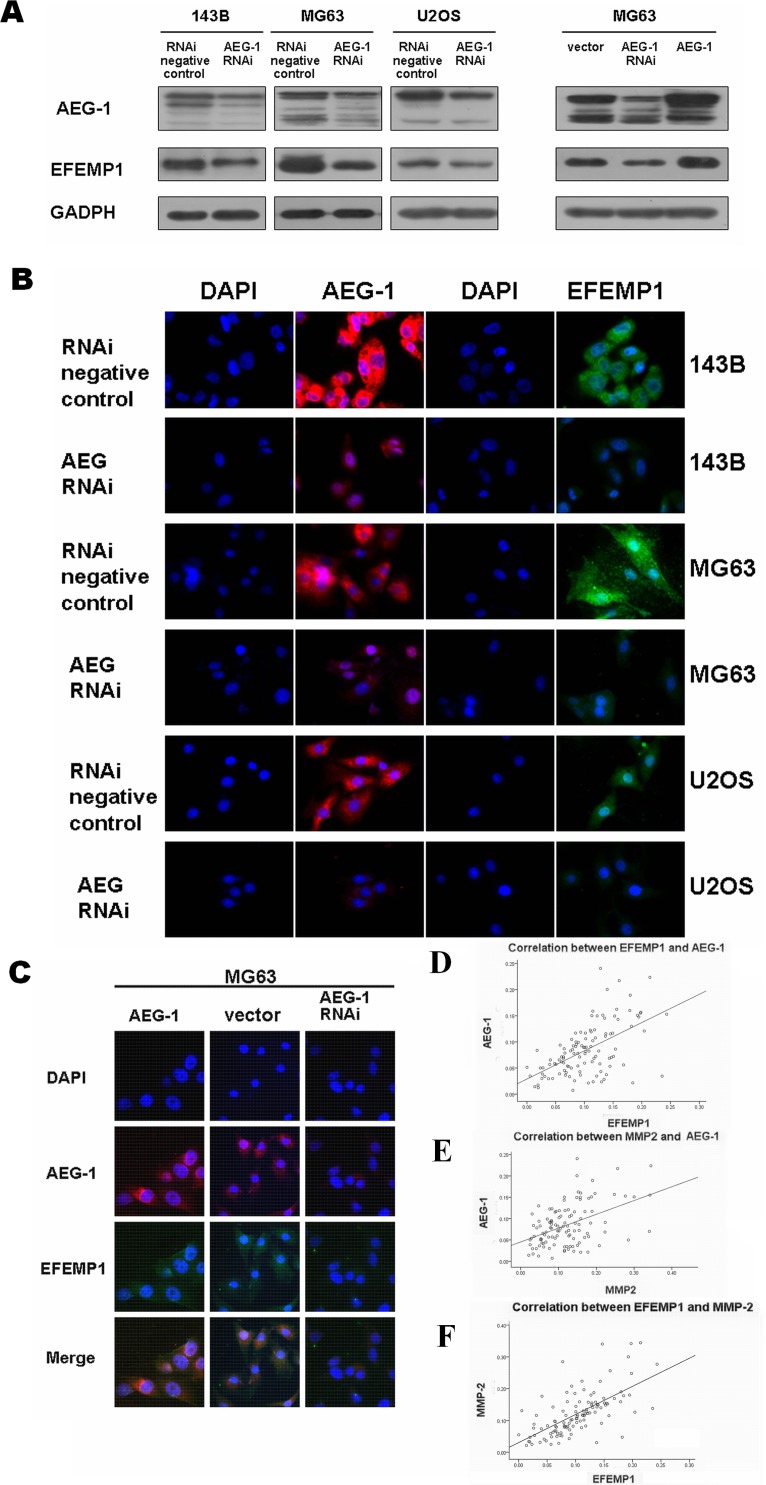
The relation between EFEMP1 and AEG-1 (**A**) Downregulation and upregulation of AEG-1 expression regulated the expression of EFEMP1 in osteosarcoma cell lines, as assessed by western blot analysis. The expression levels of AEG-1 increased after transfection of MG63 with pcDNA3.1-AEG-1 as compared with cells transfected with vector alone. Protein expression levels were normalized to GADPH. (**B**) Immunofluorescence microscopy was used to assess AEG-1 and EFEMP1 expression in osteosarcoma cell lines 143B, MG63 and U2OS after application of siRNA approaches to inhibit the endogenous expression of AEG-1. (**C**) Double immunofluorescence was performed to evaluate the colocalization of AEG-1 and EFEMP1 in MG63 cells. The expression levels of AEG-1 increased after transfection with pcDNA3.1-AEG-1. (**D**) The scatter diagram showed the strong correlation between EFEMP1 and AEG-1 expression based on Pearson's correlation analysis throught immunohistochemistry in osteosarcoma patients. (**E**) The scatter diagram showed the strong correlation between AEG-1 and MMP-2 expression based on Pearson's correlation analysis throught immunohistochemistry in osteosarcoma patients. (**F**) The scatter diagram showed the strong correlation between EFEMP1 and MMP-2 expression based on Pearson's correlation analysis throught immunohistochemistry in osteosarcoma patients.

In the present study, we examined the effects of oncogenic AEG-1 on EFEMP1 expression in osteosarcoma cells. The effects of AEG-1 downregulation and overexpression on EFEMP1 protein and RNA levels was determined by transfecting AEG-1 siRNA and AEG-1 osteosarcoma cells with pcDNA3.1-AEG-1 [16]. EFEMP1 expression was markedly induced by AEG-1 (Figure 7A, 7B and 7C), but EFEMP1 had no effect on AEG-1 expression (Figure S7).

Moreover, we added purified EFEMP1 protein to osteosarcoma cells and used siRNA techniques to inhibit the endogenous expression of AEG-1. Next, transwell migration and wound-healing assays were performed. The results showed that the migratory ability of AEG-1 siRNA osteosarcoma cells was partially recovered following treatment with EFEMP1 protein (Figure S8).

We validated the link among EFEMP1, AEG-1 and MMP-2 in osteosarcoma patients using immunohistochemical staining. There was the strong correlation between EFEMP1, AEG-1 and MMP-2 expression based on Pearson's correlation analysis, consistent with our observations *in vitro* (EFEMP1 and MMP-2, r = 0.652, P = <0.01; AEG-1 and EFEMP1, r = 0.566, P = <0.01; AEG-1 and MMP-2, r = 0.459; P = <0.01; Figure 7D-7F and Figure S9). There was no correlation between EFEMP1 and TIMP-3 as assessed by immunohistochemistry.

### EFEMP1 expression was inhibited by the selective inhibitor of NF-κB (PDTC)

**Figure 8:**
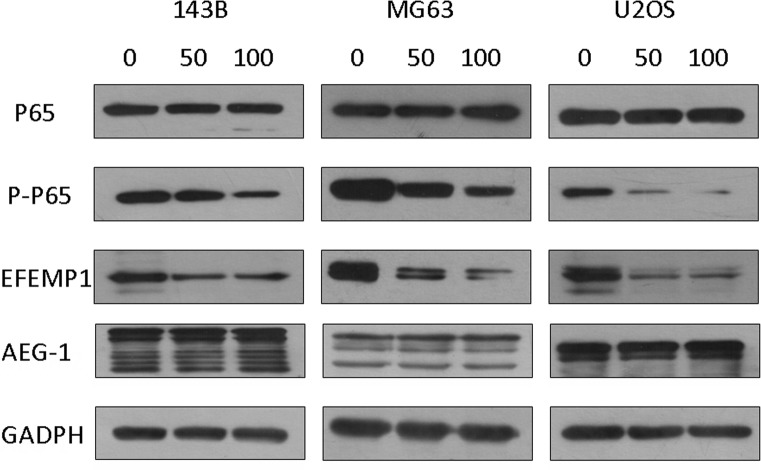
PDTC suppressed EFEMP1 expression via inhibition of NF-κB signaling pathway in osteosarcoma cells The basal level of Phospho-NF-κB p65 and EFEMP1 were decreased by treatment with 50 μM and 100 μM specific NF-κB inhibitor-PDTC in 143B, MG63 and U2OS osteosarcoma cells, without affecting NF-κB P65 and AEG-1, analyzed by western blot. Protein expression levels were normalized to GADPH.

**Figure 9:**
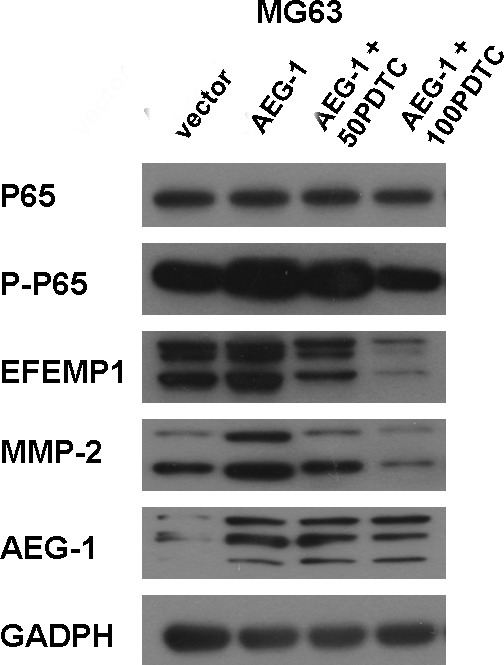
PDTC suppressed EFEMP1 expression via inhibition of NF-κB signaling pathway in osteosarcoma cells The treatment with PDTC(50 μM, 100 μM) partly suppressed the expression of Phospho-NF-κB p65, EFEMP1 and MMP-2 in osteosarcoma cells with pcDNA3.1-AEG-1 cells, without affecting NF-κB P65 and AEG-1, analyzed by western blot. Protein expression levels were normalized to GADPH.

Many published experimental results revealed AEG-1 regulates invasion and migration of tumor cells by activating the nuclear factor-κB (NF-κB) signaling pathway. To demonstrate the inhibition of the NF-κB signaling pathway could suppress EFEMP1 expression, osteosarcoma cells were pretreated with or without PDTC, and the expression levels of EFEMP1 were determined. The basal level of Phospho-p65, EFEMP1 and MMP-2 were decreased by treatment with 50μM and 100μM specific NF-κB inhibitor-PDTC for 1.5 h (Figure 8 and Figure S10). Moreover, as shown in Figure 9 the treatment with PDTC (50μM, 100μM) could markedly suppress the expression of Phospho-p65, EFEMP1 and MMP-2 in osteosarcoma cells with pcDNA3.1-AEG-1. Therefore, NF-κB pathway might be one of the effective ways which EFEMP1 was induced by AEG-1.

## DISCUSSION

A considerable body of evidence supports a role for EFEMP1 as an anti-tumor glycoprotein. According to our recent literature review, approximately 20 studies have examined the anti-tumor effects of EFEMP1. These studies included 14 kinds of malignant tumors, including lung, hepatocellular and nasopharyngeal carcinoma. For example, the levels of EFEMP1 mRNA and protein were found to be reduced in lung cancer tissues compared with normal tissues. EFEMP1 negatively modulates the invasiveness of lung cancer cells by regulating p38-MAPK and MMP-2/9/7 [17, 18]. Furthermore, EFEMP1 suppresses both the epithelial-to-mesenchymal transition and self-renewal of lung cancer stem cells by modulating the IGF1R/PI3K/AKT/GSK3β pathway [19]. EFEMP1 is downregulated in nasopharyngeal carcinomas, and its loss-of-function signiﬁcantly correlates with advanced tumor and lymph node metastasis stages by increasing the activity of phospho-AKT [20]. Anti-EGFR function of EFEMP1 had on the expression of EGFR and glioma patient prognosis[21]. Moreover, EFEMP1 blocks the migration of endothelial cells and hampers the proliferation of ﬁbrosarcoma cell lines [22].

In contrast, only about ten studies have examined the pro-tumor effects of EFEMP1, comprising only five tumor types (pancreatic carcinoma, glioma, cervical cancer, ovarian carcinoma and pleural mesothelioma). EFEMP1 was found to be highly upregulated in gliomas and enhanced substrate-specific glioma cell adhesion and motility. In addition, EFEMP1 has been shown to be overexpressed concomitantly with MMP-2, MMP-9 and ADAMTS-5 in glioma [10]. Moreover, EFEMP1 expression correlates with the expression levels of Notch-dependent genes and is a marker of Notch activation in patient-derived glioma samples [6]. EFEMP1 expression level correlates with survival in TMZ-treated glioblastoma patients [23]. EFEMP1 also promotes angiogenesis, the growth and lymph node metastasis, vascular invasion and poor prognosis of cervical carcinoma and ovarian carcinoma by upregulating the expression of VEGF[24-26].

The role of EFEMP1 in osteosarcoma has never been reported. Our results demonstrated that EFEMP1 is overexpressed in osteosarcoma patients and correlates significantly with hematogenous metastasis and poor outcomes. Concurrently, overexpression of EFEMP1 in osteosarcoma cell lines promoted migration and invasion *in vitro* and *vivo*. The results showed that EFEMP1 behaved as a pro-tumor glycoprotein in osteosarcoma and strongly suggested that EFEMP1 might be involved in the progression and metastasis of osteosarcoma. Moreover, our findings supported the tumor-promoting effects of EFEMP1. Our findings indicate that EFEMP1 can promote tumor development through a variety of mechanisms. The pro-tumor function of EFEMP1 cannot be overlooked. Future studies should examine the roles of EFEMP1 in different tumors. In addition, the dual role of EFEMP1 in tumorigenesis should be examined in greater detail.

To our knowledge, EFEMP1 is expressed in the condensing mesenchyme to produce bone and cartilage, which indicates that EFEMP1 is important for skeletal development. Compared with age-matched healthy subjects, median levels of the serum EFEMP1 proteins Fib3-1 and Fib3-2 were elevated in patients with osteoarthritis (OA) [27]. These findings strongly supported a role of EFEMP1 in organizing the development of the skeletal system, suggesting that the expression of EFEMP1 might increase during bone injury.

Epigenetic silencing of the EFEMP1 promoter by DNA methylation has been identiﬁed in many tumors, extending the anti-tumor role of EFEMP1. Inactivation of EFEMP1 by promoter methylation is associated with lung, breast, liver, endometrial, prostate and colon cancers [28-30]. Promoter methylation is the major cause of this downregulation. Stine *et al* described several genes that were hypermethylated and underexpressed, including EFEMP1 in osteosarcoma cells, compared to normal osteoblasts. These findings may differ from the present results, although methylation was not examined in the current study. However, Kresse *et al* did not evaluate patients with osteosarcoma. Moreover, the EFEMP1 promoter was found to be highly methylated in 10/19 osteosarcoma cell lines. The ratio of EFEMP1 promoter methylation was not very high [31]. Therefore, we question the conclusion of Kresse regarding the expression of EFEMP1 in osteosarcoma and propose that further research is needed.

The anti-tumor and pro-tumor activities of EFEMP1 may occur via different mechanisms. One of the mechanisms may occur via an association with MMPs. EFEMP1 is associated with decreased MMP-2 and MMP-7 levels in lung cancer [17] and MMP-2 and MMP-9 levels in endometrial carcinoma [28], which are also observed in highly metastasizing and rapidly expanding tumors. However, another interesting connection between EFEMP1 and MMPs is their simultaneously elevated expression levels. EFEMP1 can increase the expression and activity of MMP-2 and MMP-9 in malignant gliomas, a finding which is somewhat similar to the results of our study. We found a direct regulatory effect of EFEMP1 on MMP-2 expression and activity in osteosarcoma cells, and there was a strong correlation between EFEMP1 and MMP-2 expression in human osteosarcoma samples. Therefore, we proposed a possible mechanism by which EFEMP1 promotes the migration and invasion of osteosarcoma cells. Concomitantly, our results also showed that the relationship between EFEMP1 and MMP-2 was not constant and changes in different tumors.

The activity of MMP-2 is modulated at three levels: gene expression, proenzyme activation, and inhibition of hydrolytic ability by specific inhibitors. The results of the present study confirmed that EFEMP1 has a regulatory effect on MMP-2 expression. Whether the inhibitors of MMPs would work? Previous research has confirmed that EFEMP1 bind to TIMP-3 in macular degenerative diseases; they colocalize *in vivo* and form a complex *in situ* [7, 32, 33]. It has been suggested that EFEMP1 and TIMP-3 form a strong association which regulates matrix composition and affects tumor progression [34]. However, we did not find a correlation between EFEMP1 and TIMP-3 in osteosarcoma cells or in human osteosarcoma samples. These results suggested that EFEMP1 did not regulate the expression of TIMP-3 in osteosarcoma despite a strong interaction between EFEMP1 and TIMP-3. Therefore, inhibition by specific inhibitors did not play an important role in osteosarcoma progression. Moreover, the effect of the EFEMP1-TIMP-3 interaction in tumors remains unclear and requires additional research.

AEG-1 has emerged as an important oncogene. Numerous studies have documented that AEG-1 promotes tumor migration and invasion. Some findings suggest that overexpression of AEG-1 activates the NF-κB, PI3K/ Akt, MAPK, MEK, ERK1/2 and Wnt/β-catenin signaling pathways to stimulate tumor migration and invasion [35, 36]. Furthermore, AEG-1 functions as a downstream mediator of the transforming activity of oncogenic Ha-Ras and c-Myc [37]. However, how these interactions modulate tumor metastasis remains unclear. Many genes were identified as downstream target molecules of AEG-1, including activator protein 1 (AP-1) [38], tumor necrosis factor-α-related apoptosis-inducing ligand(TRAIL) [39], and aldehyde dehydrogenase 3 family member A1(ALDH3A1), hepatocyte growth factor receptor (Met) [37]. Our previous study showed that AEG-1 was overexpressed in osteosarcoma, and the expression of AEG-1 was associated strongly with invasion and poor prognosis of osteosarcoma patients [16]. In the present study, we found that EFEMP1 was not only markedly induced by AEG-1 but also promoted tumor progression including migration and invasion. This is the first study to demonstrate a relationship between EFEMP1 and AEG-1. Therefore, we speculate that EFEMP1 is a downstream target molecule of AEG-1, and EFEMP1 mediates its effects on tumor migration and invasion. Considering previous findings, we hypothesize that AEG-1 might cooperate with other transcription factor(s) because AEG-1 protein does not possess a DNA-binding domain. AEG-1 might activate the transcription of downstream target genes. We speculate that knockdown of AEG-1 affects EFEMP1 indirectly and modulates the function of several downstream target genes.

Some results suggest that AEG-1 might be important for the constitutive activation of NF-kB and AP-1, leading to enhanced cancer cell viability and invasion [38]. AEG-1 acts as a bridging factor, resulting in transcriptional activation of genes downstream of NF-κB [40]. MMPs are likely candidates for the downstream genes [11]. Our previous data strongly suggest that AEG-1 plays a crucial role in osteosarcoma progression through MMP-2 [16]. Here, we confirmed the association between EFEMP1, AEG-1 and MMP-2 in tissue specimens of osteosarcoma patients. EFEMP1 acted as an intermediate between AEG-1 and MMP-2. These results together with the findings of previous studies suggested that AEG-1 might regulate osteosarcoma invasion at least partly via the EFEMP1/MMP-2 pathway. The mechanism by which AEG-1 and EFEMP1 function together as the bridging factor to upregulate MMP-2 remains unclear. It might be a potential mechanism of AEG-1-mediated enhanced metastasis in osteosarcoma. Further investigation is needed to determine whether AEG-1 interacts with EFEMP1 or other factors to activate MMP-2.

Some researches documented that AEG-1 might function as a bridging factor facilitating interaction among p50-p65, CBP, and the basal transcription machinery [41]. Therefore AEG-1 functions as a coactivator in regulating NF-κB–mediated transcription, consequently augmenting expression of genes necessary for invasion of malignant tumor cells [41]. AEG-1 could enhance the binding of p65 to the MMP-1 promoter [42], thereby activate downstream genes by functioning as a linker between p65 and CBP to form the basal transcriptional machinery. Now our studies showed that AEG-1 could regulate the expression of EFEMP1 in osteosarcoma cells. Moreover, EFEMP1 expression was inhibited by the selective inhibitor of NF-κB (PDTC) in osteosarcoma cells. This possible transcription machinery about the expression of EFEMP1 by NF-κB pathways and the relation between EFEMP1 and AEG-1 had not reported in the past. Based the nuclear translocation in mediating NF-κB activation by AEG-1, we thought that NF-κB pathways may be one of effective ways which EFEMP1 was induced by AEG-1. These finding are new and important. We look forward to these finding to help to illuminate the functional machinery of EFEMP1 and AEG-1.

In conclusion, our results showed that EFEMP1 was specifically upregulated in osteosarcoma, potentially contributing to the migratory and invasive capability of osteosarcoma cells. EFEMP1 is not only a poor prognostic indicator in osteosarcoma, but it is also a candidate for molecular targeted therapy. EFEMP1 upregulates the expression of MMP-2. It illustrats a new mode of action for the molecular mechanism underlying the invasiveness of osteosarcoma. This mechanism was not associated with TIMP-3. Together with our previous results, we speculated that EFEMP1 is a downstream target molecule of AEG-1, mediating the tumor migration and invasion. Moreover, we thought that NF-κB pathways may be one of effective ways which EFEMP1 was induced by AEG-1. Another mechanism by which AEG-1 promotes metastasis might include an indirect enhancement of MMP-2 expression via activation of EFEMP1 by NF-κB signaling pathway.

## MATERIALS AND METHODS

### Tissue specimens

Tissue specimens and controls were obtained from patients who underwent surgery at the First Affiliated Hospital at Sun Yat-sen University. Parafﬁn-embedded tissue blocks were retrieved from 113 osteosarcoma patients, 10 normal bone tissues, 10 osteoblastoma specimens, 10 osteoma specimens, 6 osteoid osteoma specimens and 12 tumor-like bone lesions (osteofibrous dysplasia and fibrous dysplasia). Fresh osteosarcoma tissues and adjacent normal tissue samples were obtained at the time of surgical resection and immediately frozen to −80°C until use. The use of tissue specimens in research was approved by the research ethics committee of the First Affiliated Hospital at Sun Yat-sen University.

These osteoblastoma patients received standard neoadjuvant chemotherapy followed by resection of the tumor and postoperative chemotherapy at the Musculoskeletal Tumor Center of Sun Yat-Sen University (Guangzhou, People's Republic of China). Clinicopathologic features of the 113 patients are shown in Table 1.

### Cell culture and treatment

The human osteosarcoma cell lines MG63, U2OS and 143B were obtained from Cell Banks at the department of Pathology, Zhongshan School of Medicine, Sun Yat-sen University, Guangzhou, China. Cells were cultured in Minimum Essential Medium (Invitrogen, Grand Island, NY) or Dulbecco's Modiﬁed Eagle's Minimum (Invitrogen, Grand Island, NY) supplemented with 10% (v/v) fetal bovine serum (Invitrogen), 100 μg/μL streptomycin and 100 μg/μL penicillin in a 37°C incubator containing 5% CO_2_.

To examine the effect of exogenous EFEMP1 protein stimulation on the migration and invasion of osteosarcoma cells, EFEMP1 (Human) recombinant protein(Abnova, Taiwan) was added at final concentrations of 25, 50, 100, and 200 ng/mL.

To evaluate the role of NF-κB signaling pathway in the induction of EFEMP1 by AEG-1, Ammonium pyrrolidinedithiocarbamate(PDTC), a selective inhibitor of NF-κB (Sigma-Aldrich, United States) was dissolved in pure water and used at final concentrations of 50 or 100 μM.

### Stable cell lines and plasmids

Two osteosarcoma cell lines(143B, U2OS) were selected to generate stable cell lines in this study. The retroviral packaging system was purchased from Clontech. For EFEMP1 over-expression, ectopic EFEMP1 coding sequence was amplified by polymerase chain reaction (PCR). The amplified product was cloned into the pBaBb-puromycin plasmid and confirmed by sequencing. Osteosarcoma cell lines were transfected with aforementioned constructed plasmids or empty vector. Stably transfected cell lines were selected with 0.5 μg/ml puromycin at 48 hours after infection.

### siRNA

Cells were seeded in 6-well plates at a density of 1 × 10^5^ cells per well. Cationic lipid complex was prepared by incubating 50 nM siRNA with 5 μL of Lipofectamine® RNAiMAX Transfection Reagent (Invitrogen, Carlsbad, CA) in 500 μL of Opti-MEM® I Reduced Serum Medium (Invitrogen, Carlsbad, CA) for at least 20 min and added to the cells medium. After a 6-h incubation, the medium was replaced with fresh medium. The cells were harvested at 24 h~72 h after transfection for analysis. The EFEMP1 siRNA-1 5′-GCAAUGCACUGACGGAUAUdTdT-3′ and 3′-dTdT CGUUACGUGACUGCCUAUA-5′ and the EFEMP1 siRNA-2 5′ –GAGUUCUACCUACGACAAAdTdT-3′ and 3′-dTdT CUCAAGAUGGAUGCUGUUU-5′ were synthesized by Ribobio (Guangzhou, China). For the AEG-1 siRNA, a previously described sequence was used. Control siRNA was provided by Ribobio (Guangzhou, China).

### RNA extraction and quantitative real-time PCR

Total RNA was extracted using TRIzol reagent (Invitrogen, Carlsbad, CA) according to the manufacturer's instructions. Reverse-transcription was performed using the RevertAid First Strand cDNA Synthesis Kit (Roche Diagnostics, Mannheim, Germany). Quantitative real-time PCR (qRT-PCR) was performed using FastStart Universal SYBR Green Master (ROX) (Roche Diagnostics, Mannheim, Germany) with a StepOne fast real-time PCR system (Applied Biosystems). Target gene expression was calculated using ΔΔCt and comparative methods after normalization to GAPDH expression.

### Western blotting

Standard western blotting was conducted for the protein expression analyses. The protein contents of cleared lysates were determined using a BCA Protein Quantitative Analysis Kit (CoWin Biotech Co., Ltd., China). The membranes were incubated with primary antibodies overnight at 4°C and then with the appropriate secondary antibody. The following primary antibodies were used: EFEMP1 antibody (AP9095a, ABGENT, San Diego, CA), MMP-2 antibody (AM1844a, ABGENT, San Diego, CA), AEG-1 antibody (H00092140-D01, Abnova, Taiwan), NF-κB P65 antibody (6959, Cell Signaling, CA) and Phospho-NF-κB p65 antibody (3033, Cell Signaling, CA).

### Histology, immunohistochemistry and immunofluorescence analysis

Normal and tumor tissue samples were selected by two pathologists based on the diagnosis and microscopic morphology. Standard immunohistochemistry of sections from parafﬁn-embedded tissue blocks were conducted. Briefly, the sections were deparaffinized in xylene and rehydrated using a series of graded alcohols. The sections were then treated with 3% hydrogen peroxide for 10 minutes to exhaust endogenous peroxidase activity. The antigens were retrieved in 0.01 M sodium citrate buffer (pH 6.0) using a microwave oven. The sections were incubated overnight using a primary antibody in a humidified container at 4°C, after 30 minutes of preincubation in 10% normal goat serum to prevent nonspecific staining. The negative control was used by PBS instead of the primary antibody. The sections were incubated with EnVision-HRP secondary antibody (Dako, Carpinteria, CA) for 30 minutes at room temperature. Then, the tissue slides were treated with a nonbiotin horseradish peroxidase detection system according to the manufacturer's instructions.

Standard immunofluorescence of the cell climbing pieces was carried out. Briefly, the pieces were fixed in paraformaldehyde. The pieces were permeabilized with 0.5% Triton X-100 in PBS for 15 minutes. The pieces were then blocked for 30 minutes at room temperature with 5% BSA in PBS and incubated with a primary antibody overnight at 4°C in a humidified container. The pieces were then rinsed in PBS and incubated with Dylight488–conjugated anti-mouse or Dylight 549–conjugated anti-rabbit IgG (EarthOx, California, USA), respectively, for 1 hour at 37°C. The pieces were stained with DAPI (Beyotime, China) solution for 5 min at room temperature, then washed using PBS for 10 min. Images were analyzed using an immunofluorescence microscope.

The following primary antibodies were used: EFEMP1 antibody (AP9095a, ABGENT, San Diego, CA), MMP2 antibody (AM1844a, ABGENT, San Diego, CA), and AEG-1 antibody (H00092140-D01, Abnova, Taiwan). Immunohistochemical expression was evaluated using Image-Pro Plus 6.0 software. In brief, ﬁve positive expression ﬁelds in a section were selected at random, and then the positive regions were analyzed in Image-Pro Plus 6.0 to determine the integral optical density and area. The average optical density was calculated. The average of ﬁve optical density values was determined to represent the expression intensity in the section.

### Colony formation assay

Cells were trypsinized, counted, and seeded for the colony-forming assay in 60-mm dishes at a density of 200 cells per dish. Colonies containing >50 cells were scored after 14 days by stained with crystal violet.

### Matrigel invasion assays

Matrigel invasion was measured using transwell cell culture chambers according to the manufacturer's manual. We used 24-well BioCoat cell culture inserts (BD) with an polyethylene terephthalate membrane (8-μm porosity) coated with Matrigel Basement Membrane Matrix (100 μg/cm^2^; BD). 0.5~2 × 10^4^ cells were placed into the top chamber of each insert and incubated at 37°C for 24~48 hours, according to the invasion ability of different cell lines.

### Gelatin zymography

Gelatinase activity was determined by zymography. In brief, serum-free medium was collected from the cell culture, concentrated (Amicon® Ultra Centrifugal Filters, Merck Millipore) and submitted to 10% polyacrylamide gel electrophoresis with 1% gelatin as a substrate in an ice bath (Applygen Technologies, China). Gels were incubated overnight at 37°C in renaturing and developing buffers, stained with Coomassie brilliant blue R-250 and destained in glacial acetic acid and methanol.

### *In vitro* wound-healing assays

In brief, cells were cultured in 6-well plates in medium without fetal bovine serum. A wound was made across the well with a pipette tip. The wound was photographed immediately and again at 6 h, 12 h, 24 h, 36h and 48 h after scraping to document cellular migration across the wound. The migration distance was measured using Image-Pro Plus 6.0 software.

### Tumorigenicity assays

For tumorigenicity assays, osteosarcoma cell lines 143B cells that were stably transfected with empty vector, EFEMP1 were injected near the axillary fossa subcutaneously (1 × 10^6^ cells in 150 μL of PBS per BALB/c-nu mouse, 4- to 5-weeks old, male; 8 mice per group), and the mice were monitored for 30 days. When length of the tumor was more than 2.0 cm, the experiment was stopped and the mice were killed by cervical dislocation, according to the protocol developed by the Guidance of Institutional Animal Care and Use Committee at Sun Yat-Sen University. Subcutaneous transplantation tumor, lungs and livers were detected by macrography and histological examination in the final experiment.

### Experimental metastasis assay

Male Balb/c nude mice (4- to 5- weeks old) were used, and each experimental group (empty vector-143B, and EFEMP1-143B) consisted of 8 mice. Briefly, 5 × 10^5^ cells were injected intravenously through the tail vein into each mouse. All mice were euthanized 4 weeks by cervical dislocation. The presence of tumor nodules was macroscopically determined, and the number of tumor nodules formed on the lung and liver surfaces was counted. The livers and lungs were excised and embedded in paraffin. All animal procedures were performed in full accordance with the protocol developed by the Guidance of Institutional Animal Care and Use Committee at Sun Yat-Sen University.

### Statistical analysis

Data between groups were evaluated using a two-tailed Student's t-test or one-way analysis of variance. Spearman's rank correlation coefficients were computed to assess the relationship between different factors. For all comparisons, a *P*-value <0.05 was considered statistically signiﬁcant unless otherwise indicated. Kaplan-Meier survival curves were compared using the log-rank test.

## SUPPLEMENTARY INFORMATION FIGURES


