# Supplementary material for: EFEMP1 promotes the migration and invasion of osteosarcoma via MMP-2 with induction by AEG-1 via NF-κB signaling pathway
Source: Oncotarget. 2015 Mar 29;6(16):14191–208. doi: 10.18632/oncotarget.3691 (PMC4546460; doi:10.18632/oncotarget.3691)
Supplement: Supplementary file 1 [file oncotarget-06-14191-s001.pdf]

EFEMP1 promotes the migration and invasion of osteosarcoma via MMP-2 with induction by AEG-1 via NF-κB signaling pathway

Supplementary Information

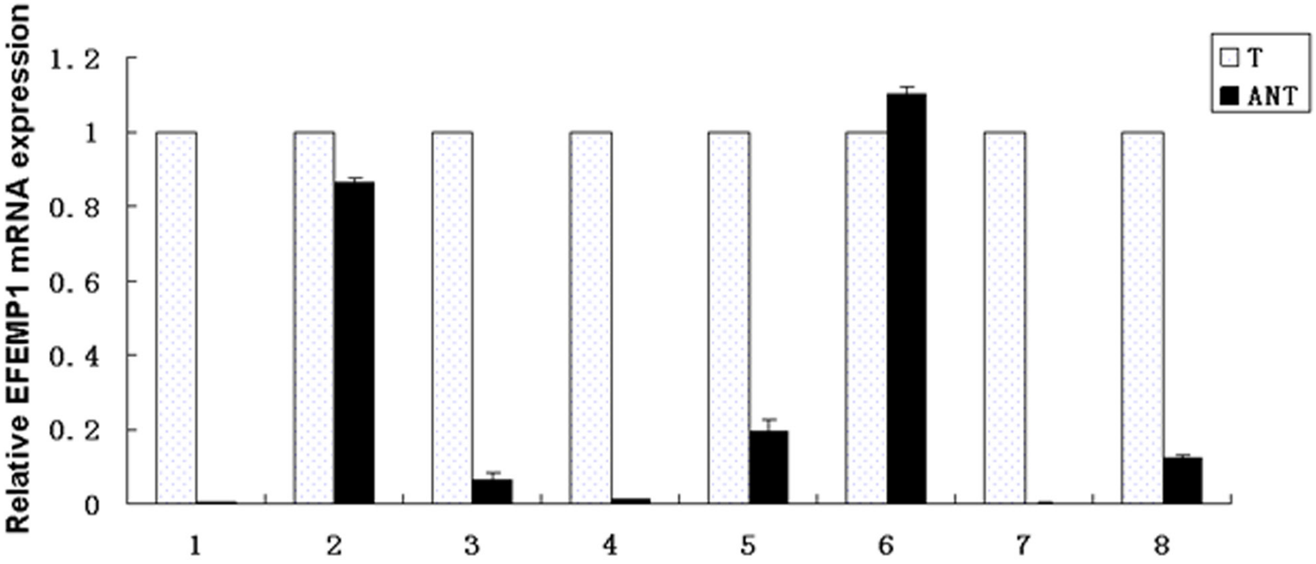

Figure S1: Comparative analysis of EFEMP1 mRNA levels in osteosarcoma tumor (T) and paired adjacent non-tumor tissues (ANT). Comparative quantification of EFEMP1 mRNA in paired primary osteosarcoma tissues (T) and adjacent non-tumor tissues (ANT), with each pair obtained from the same patient.

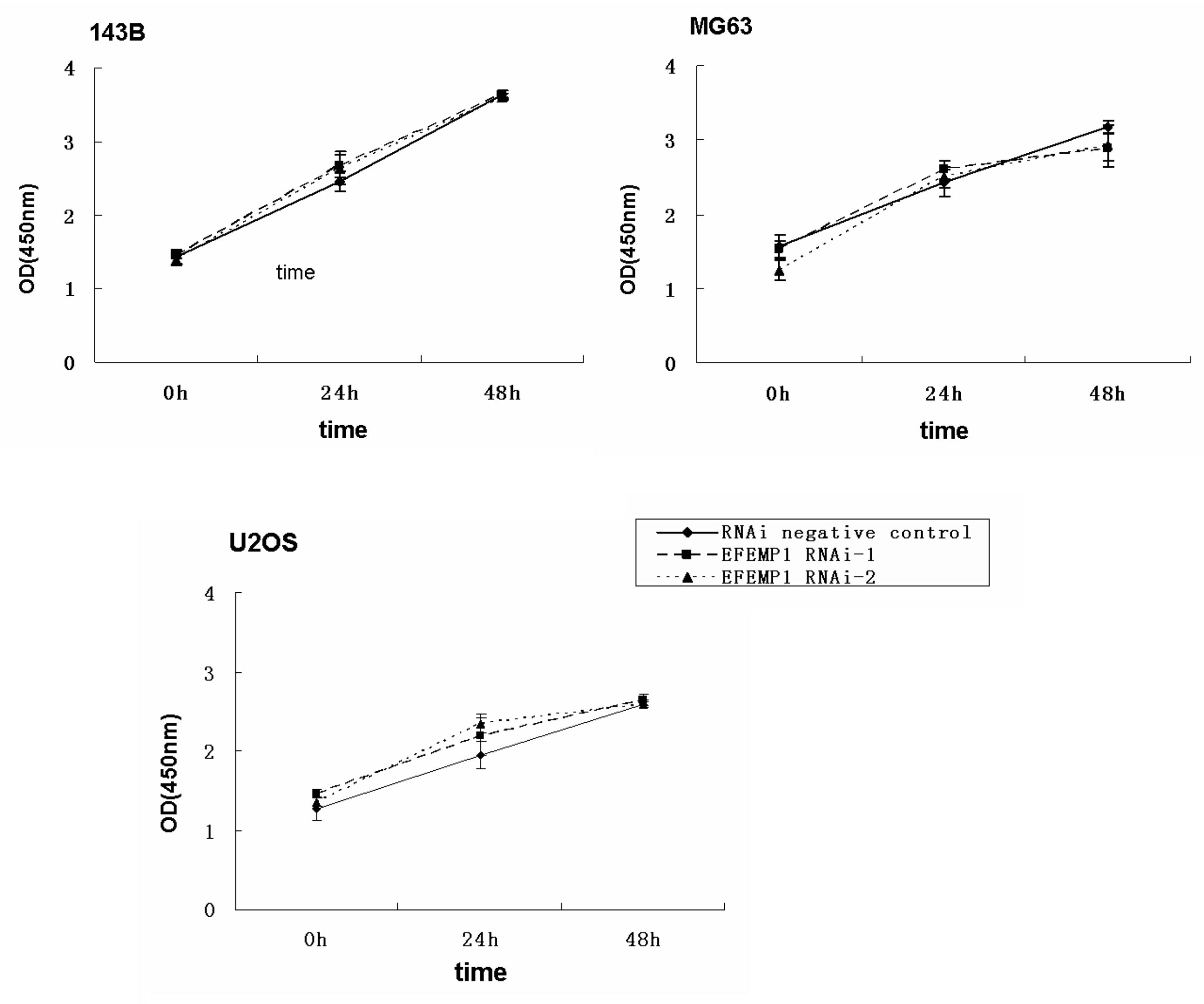

Figure S2: Effect of EFEMP1 expression on the proliferation rate of osteosarcoma. The CCK8 assay showed that the proliferation rate was not significantly different between the negative control and cells with reduced EFEMP1 expression during a 48 h period.

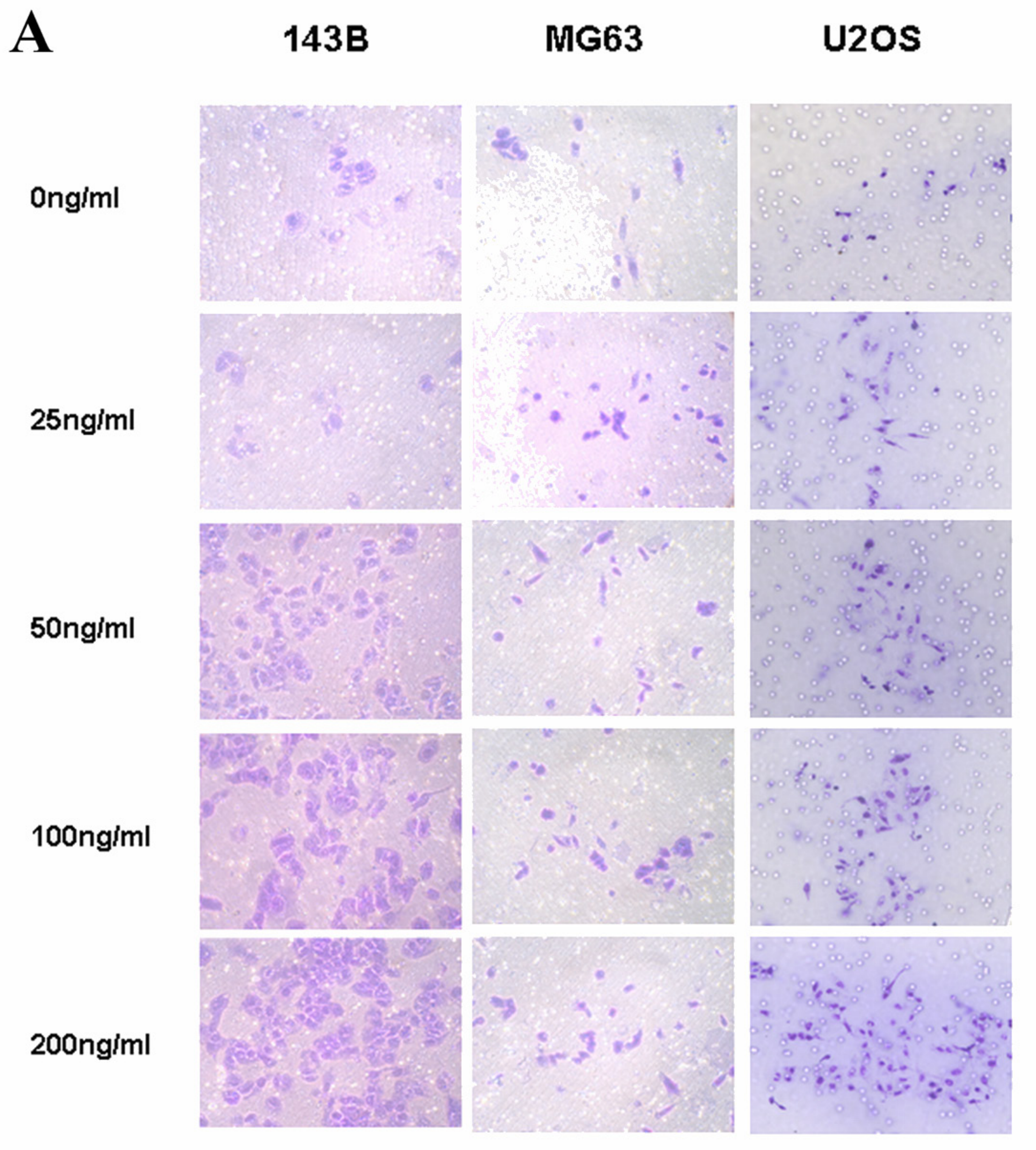
A
143B
MG63
U2OS
0ng/ml
25ng/ml
50ng/ml
100ng/ml
200ng/ml

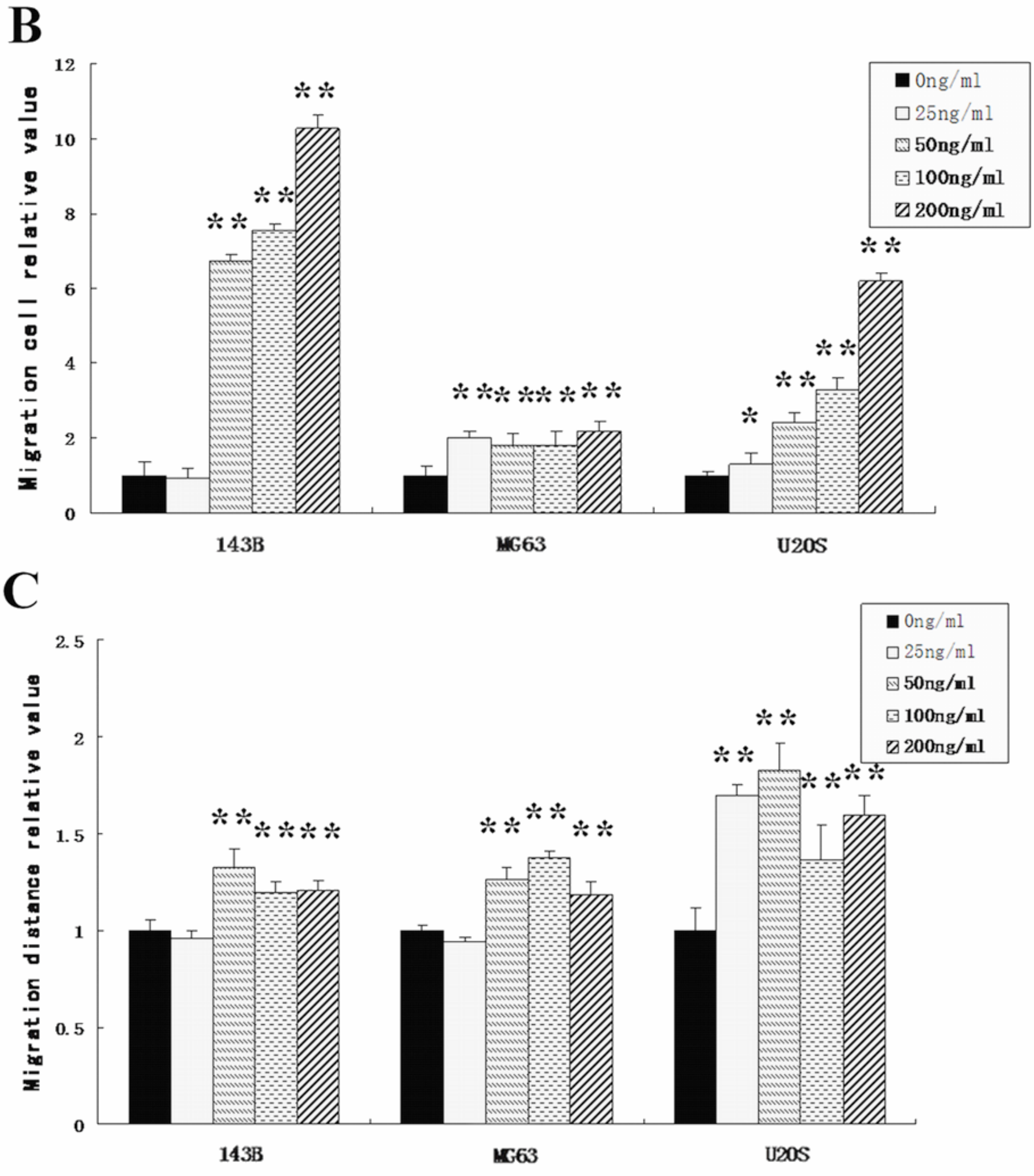
B
Migration cell relative value
0
2
4
6
8
10
12
0ng/ml
25ng/ml
50ng/ml
100ng/ml
200ng/ml
143B
MG63
U2OS
C
Migration distance relative value
0
0.5
1
1.5
2
2.5
0ng/ml
25ng/ml
50ng/ml
100ng/ml
200ng/ml
143B
MG63
U2OS

Figure S3: Exogenous overexpression of EFEMP1 promoted the invasion of osteosarcoma cell lines *in vitro*. (A) The invasive ability of osteosarcoma cell after treatment with EFEMP1 protein (25, 50, 100, and 200 ng/mL) increased compared with the negative controls. The 143B (0.5×10^4), MG63 (1×10^4) and U2OS (2×10^4) cells were added to the upper chamber in serum-free medium. Migrating cells were scored using a microscope at 400× magnification. (B) Relative quantification of penetrated cells in lower chamber represents the mean of three different experiments. (C) The migratory ability of osteosarcoma cells after treatment with EFEMP1 protein (25, 50, 100, and 200 ng/mL) increased compared with the negative controls. * versus control, $P < 0.05$, ** versus control, $P < 0.01$.

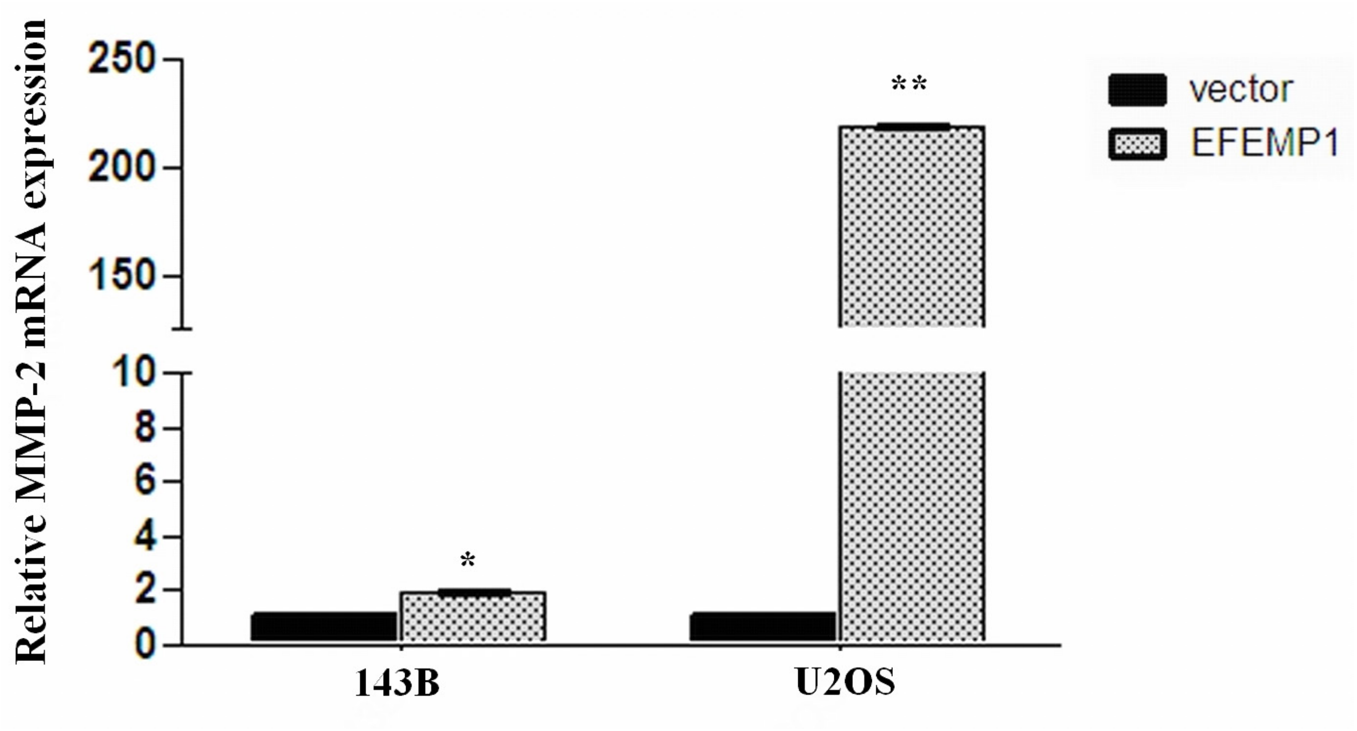

Figure S4: Changes in MMP-2 mRNA levels were quantified in osteosarcoma cell lines 143B and U2OS stably transfected with EFEMP1 expression plasmid. The mRNA expression levels are presented as the increasing fold compared with the empty vector control cells and were normalized to GAPDH.

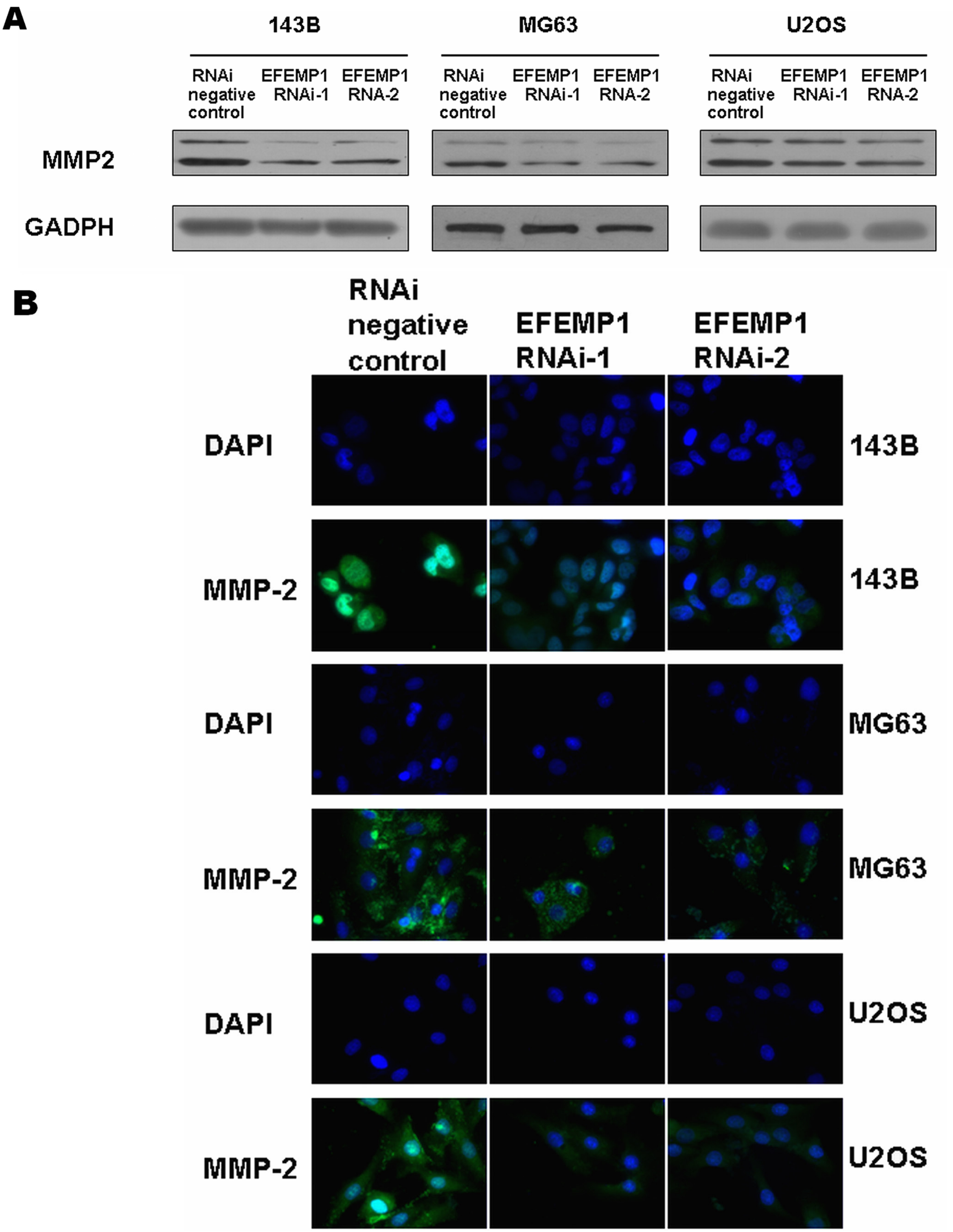
A
143B
MG63
U2OS
RNAi negative control
EFEMP1 RNAi-1
EFEMP1 RNA-2
MMP2
GADPH
B
RNAi negative control
EFEMP1 RNAi-1
EFEMP1 RNAi-2
DAPI
MMP-2
143B
MG63
U2OS

C

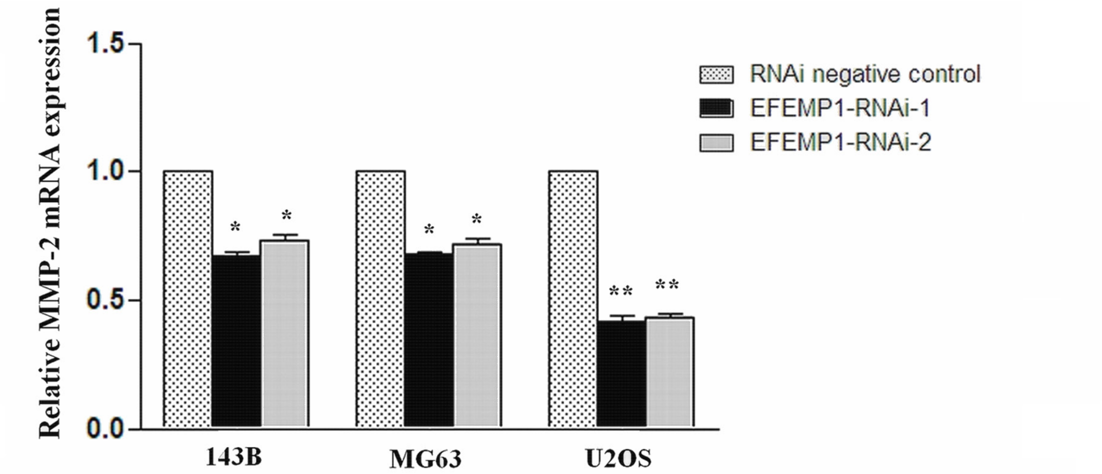

D

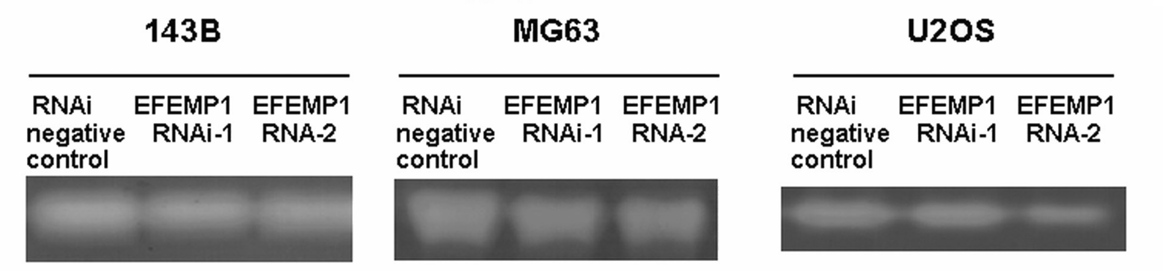

Figure S5:The expression of MMP-2 decreased after application of EFEMP1 siRNA in osteosarcoma cells. (A) Downregulation of EFEMP1 expression by siRNA regulate the expression of MMP-2 in osteosarcoma cell lines 143B, MG63 and U2OS with western blot analysis. Protein expression levels were normalized with GADPH. (B) Evaluated MMP-2 by immunofluorescence microscopy in osteosarcoma cell lines 143B, MG63 and U2OS after small interfering RNA approaches to inhibit the endogenous expression of EFEMP1. (C) Changes in MMP-2 mRNA levels were quantified in EFEMP1 RNAi–transduced osteosarcoma cells. The mRNA expression levels are presented as increasing fold compared with the negative control cells, and the values were normalized to GAPDH. (D) Conditioned media harvested from treated osteosarcoma cells were analyzed by gelatin zymography. The white bands represent MMP-2-mediated gelatin digestion. It is showed that MMP-2 activity was abolished in EFEMP1- suppressing osteosarcoma cells.

A

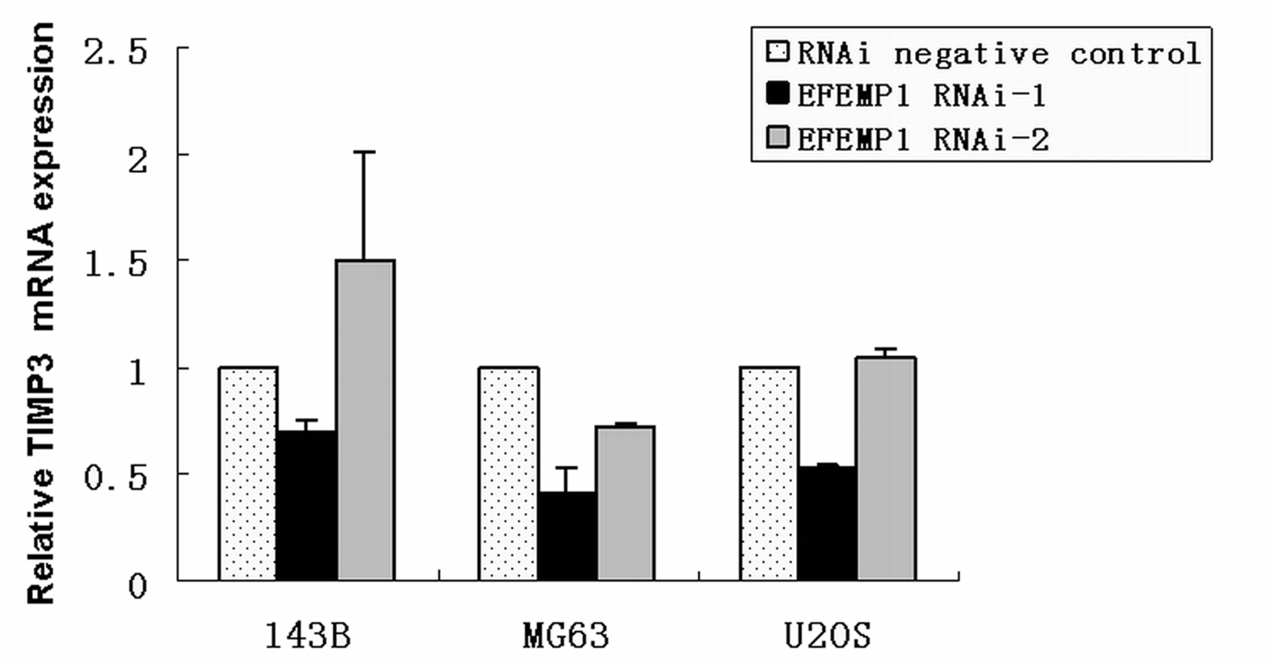

Relative TIMP3 mRNA expression
2.5
2
1.5
1
0.5
0
143B
MG63
U2OS
RNAi negative control
EFEMP1 RNAi-1
EFEMP1 RNAi-2

B

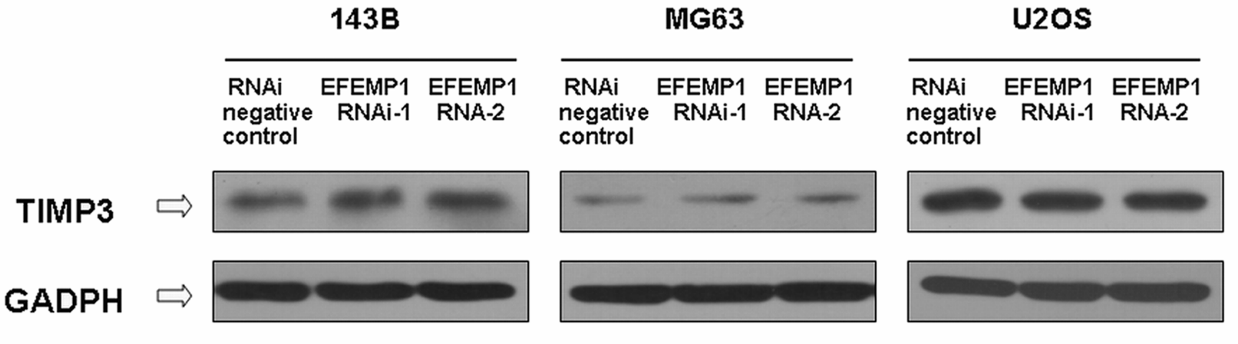

143B
MG63
U2OS
RNAi negative control
EFEMP1 RNAi-1
EFEMP1 RNA-2
TIMP3
GADPH

c

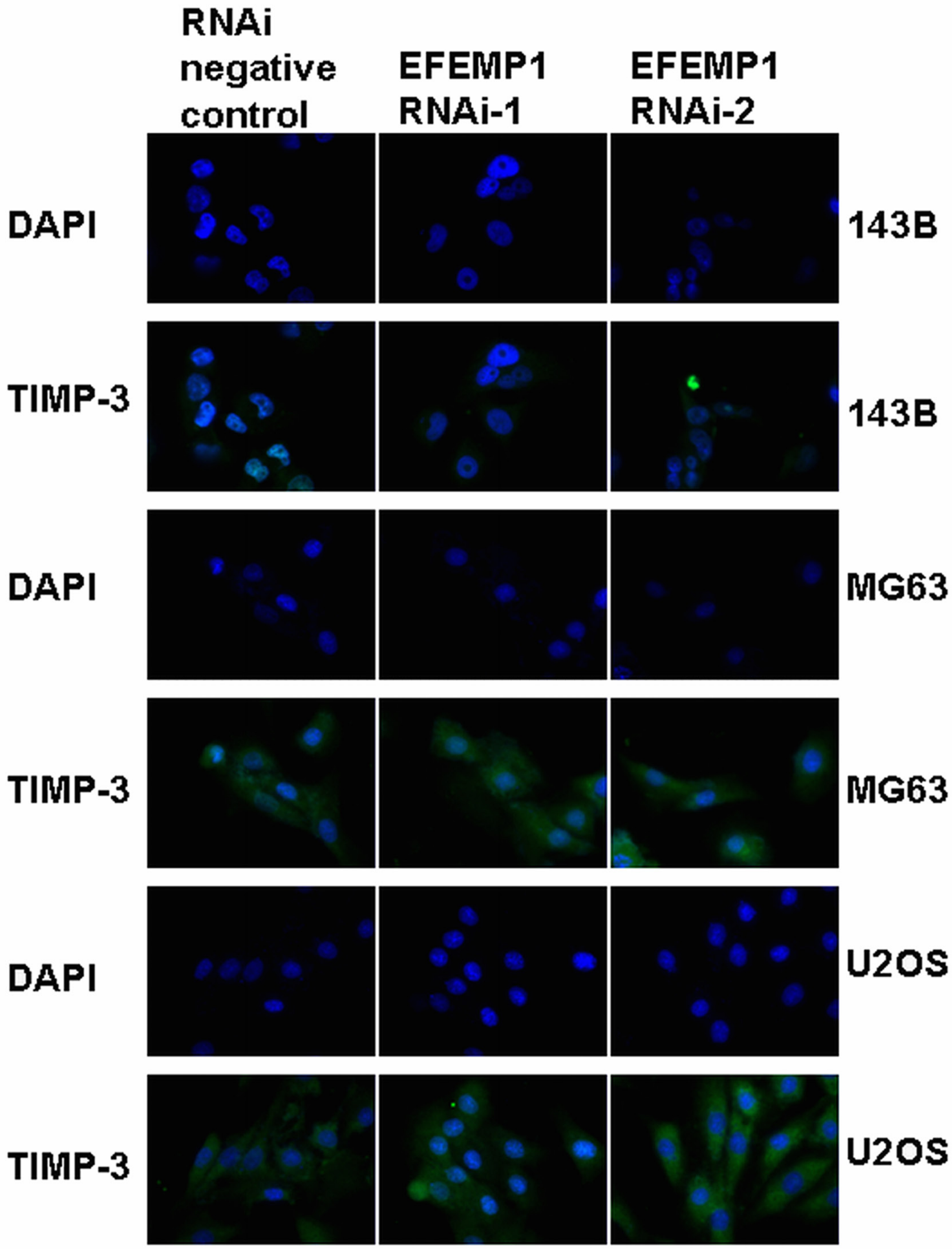
RNAi negative control
EFEMP1 RNAi-1
EFEMP1 RNAi-2
DAPI
TIMP-3
DAPI
TIMP-3
DAPI
TIMP-3
143B
143B
MG63
MG63
U2OS
U2OS

Figure S6: EFEMP1 did not associate with TIMP-3. (A) Changes in TIMP3 mRNA levels were quantified in EFEMP1 RNAi–transduced osteosarcoma cells. The mRNA expression levels are presented as increasing fold compared with the negative control cells, and the values were normalized to GAPDH. (B) Expression of EFEMP1 in the RNAi negative control (left) and EFEMP1 knockdown (right) osteosarcoma cell lines 143B, MG63 and U2OS, as analyzed by western blot using an anti–TIMP-3 antibody. Protein expression levels were normalized to GADPH. (C) Immunofluorescence microscopy was used to assess TIMP-3 in osteosarcoma cell lines 143B, MG63 and U2OS after application of siRNA approaches to inhibit the endogenous expression of EFEMP1.

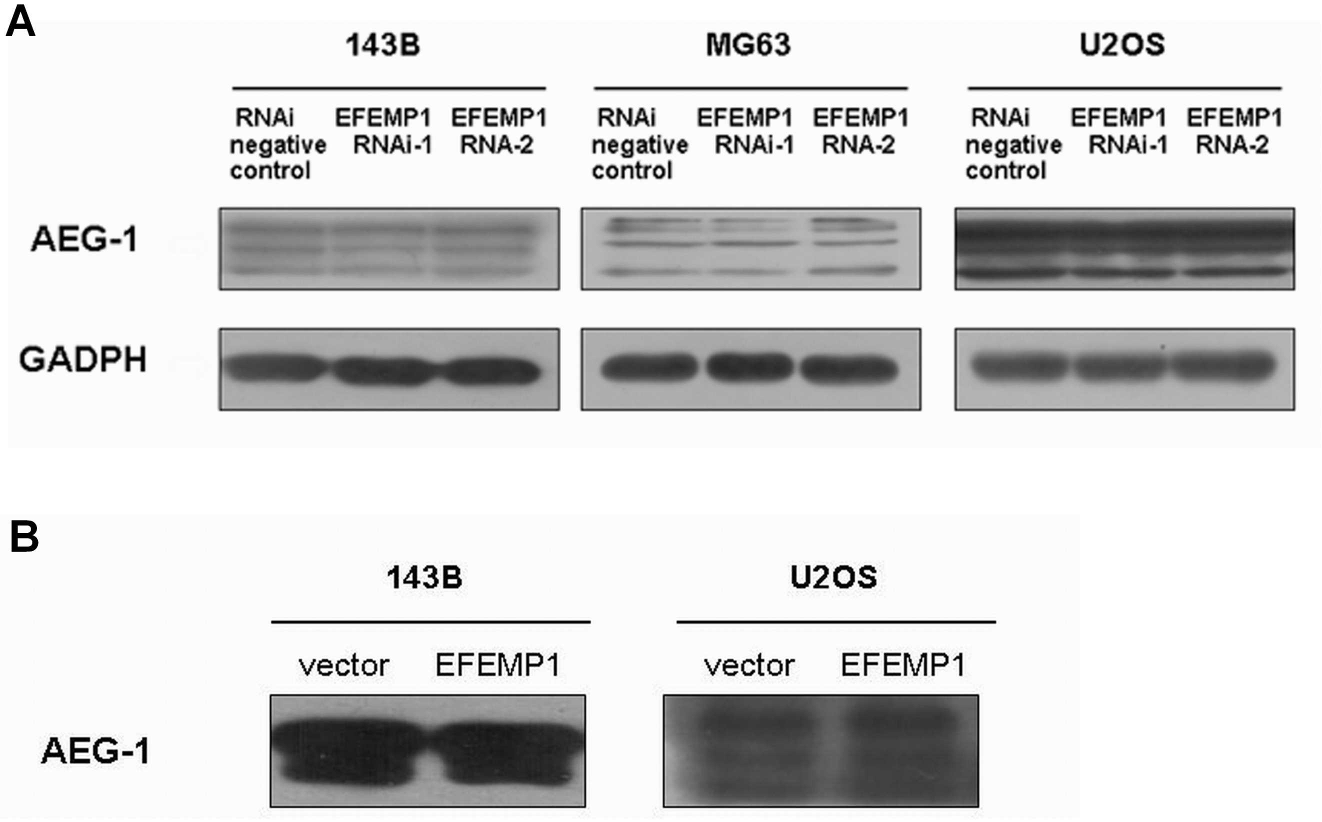

Figure S7: Effect of EFEMP1 on AEG-1. (A) Downregulation of EFEMP1 expression by siRNA did not regulate the expression of AEG-1 in osteosarcoma cell lines 143B, MG63 and U2OS based on western blot analysis. Protein expression levels were normalized to GADPH. (B) AEG-1 protein levels was not different between 143B and U2OS cells stably transfected with EFEMP1 expression plasmid with the empty vector control groups, as assessed by western blot analysis.

A

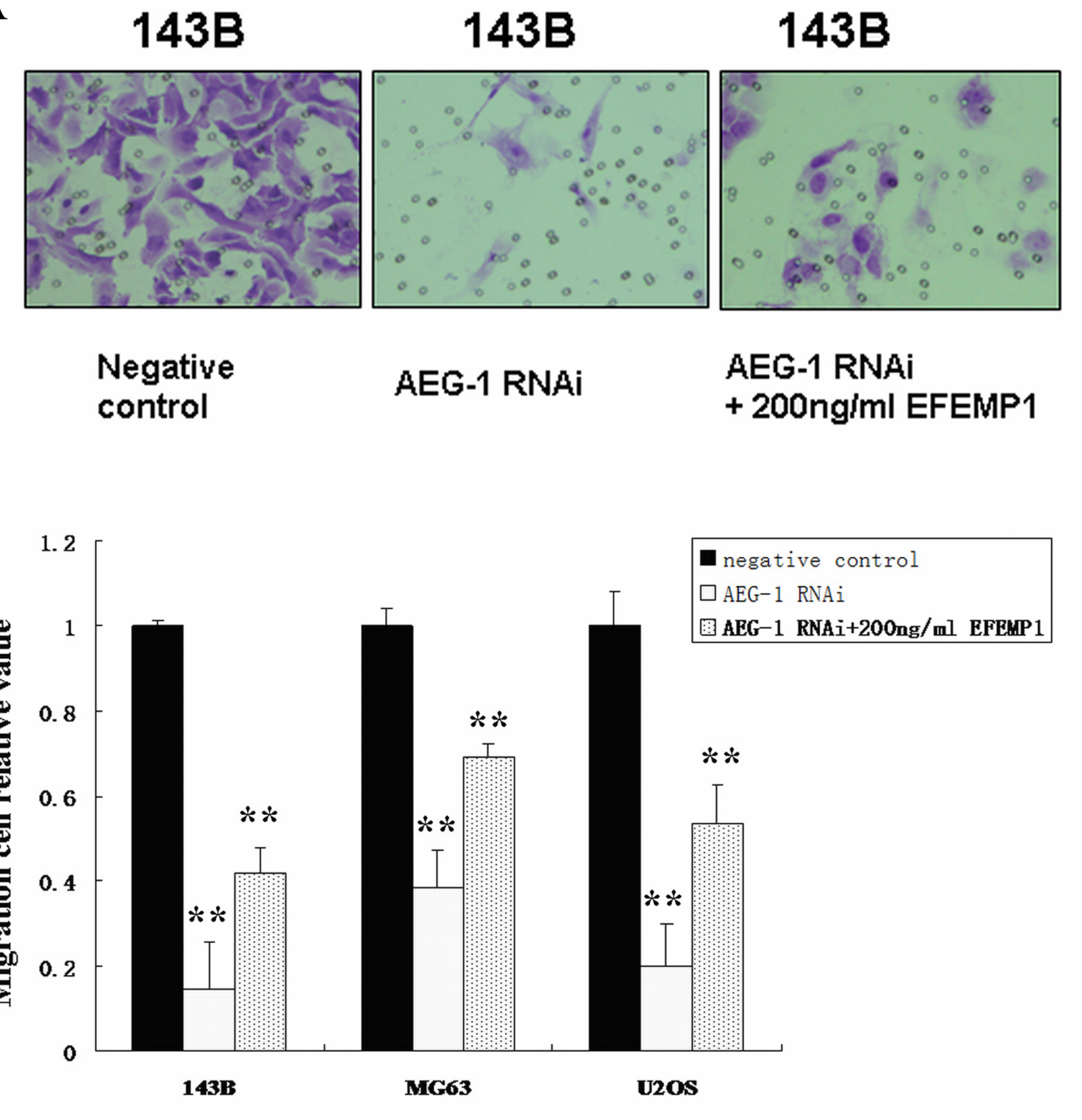
143B
143B
143B
Negative control
AEG-1 RNAi
AEG-1 RNAi + 200ng/ml EFEMP1
negative control
AEG-1 RNAi
AEG-1 RNAi+200ng/ml EFEMP1
Migration cell relative value
1.2
1
0.8
0.6
0.4
0.2
0
**
**
**
**
**
**
143B
MG63
U2OS

B

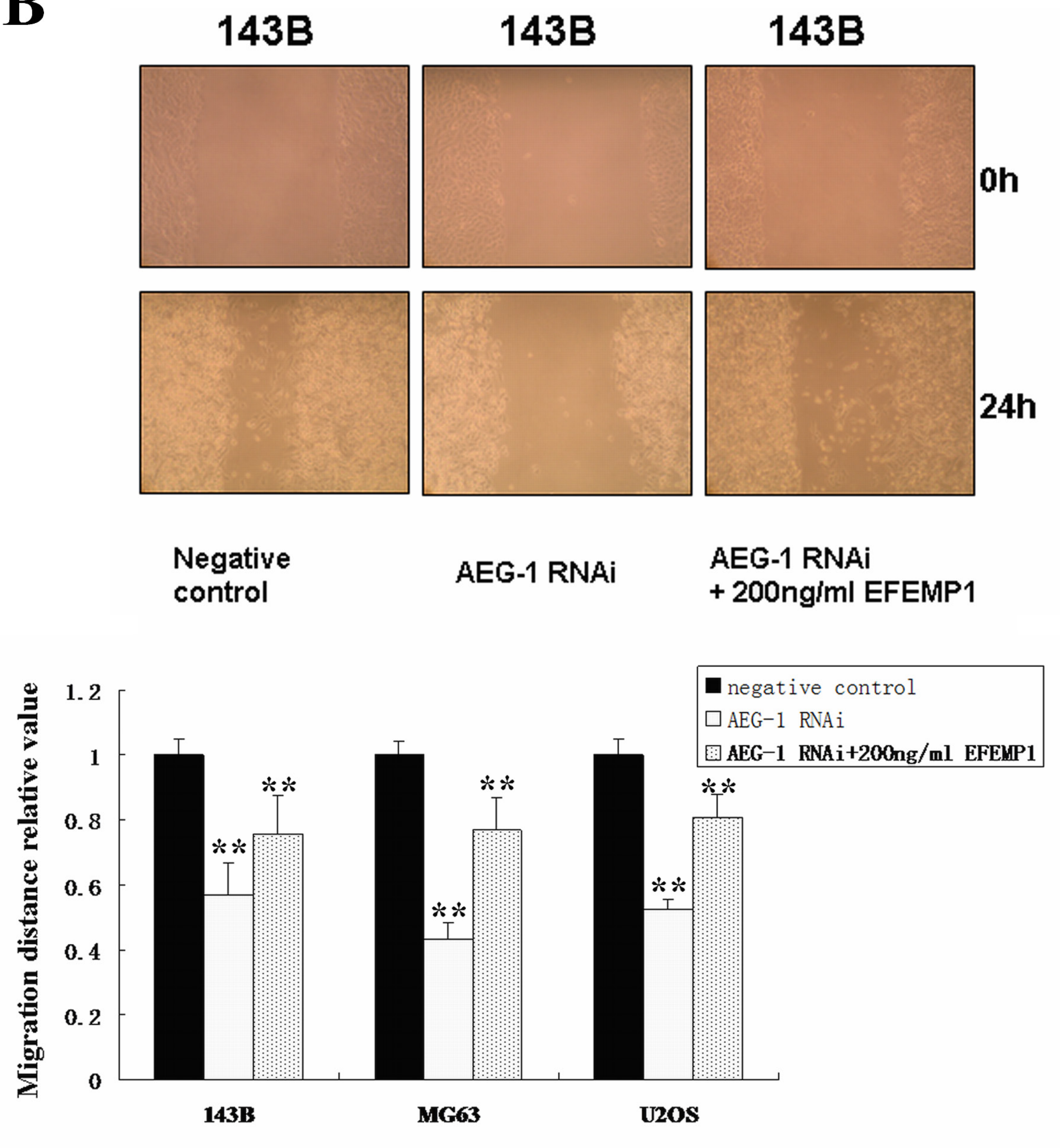
143B
143B
143B
0h
24h
Negative control
AEG-1 RNAi
AEG-1 RNAi + 200ng/ml EFEMP1
negative control
AEG-1 RNAi
AEG-1 RNAi+200ng/ml EFEMP1
Migration distance relative value
1.2
1
0.8
0.6
0.4
0.2
0
**
**
**
**
**
**
143B
MG63
U2OS

Figure S8: Application of purified EFEMP1 protein recovered the migratory ability of AEG-1 siRNA osteosarcoma cells. (A) Reduced AEG-1 expression inhibited the invasive ability of osteosarcoma cells and addition of purified EFEMP1 protein partially reversed this effect. Representative pictures are shown of penetrated 143B cells in lower chamber (upper) and quantification of the indicated cells (lower) was performed using the transwell matrix penetration assay. The 143B (2×10^4), MG63 (2×10^4) and U2OS (2×10^4) cells were added to the upper chamber in serum-free medium. Migrating cells were scored using a microscope at 400× magnification. Quantification of the penetrated cells represents the mean of three independent experiments. (B) Wound-healing assays in osteosarcoma cells showed that downregulation of AEG-1 significantly inhibited cell migration compared with the control group. Addition of purified EFEMP1 protein partially recovered this effect. The relative migration distance is shown in a bar chart. * versus control, $P < 0.05$, ** versus control, $P < 0.01$.

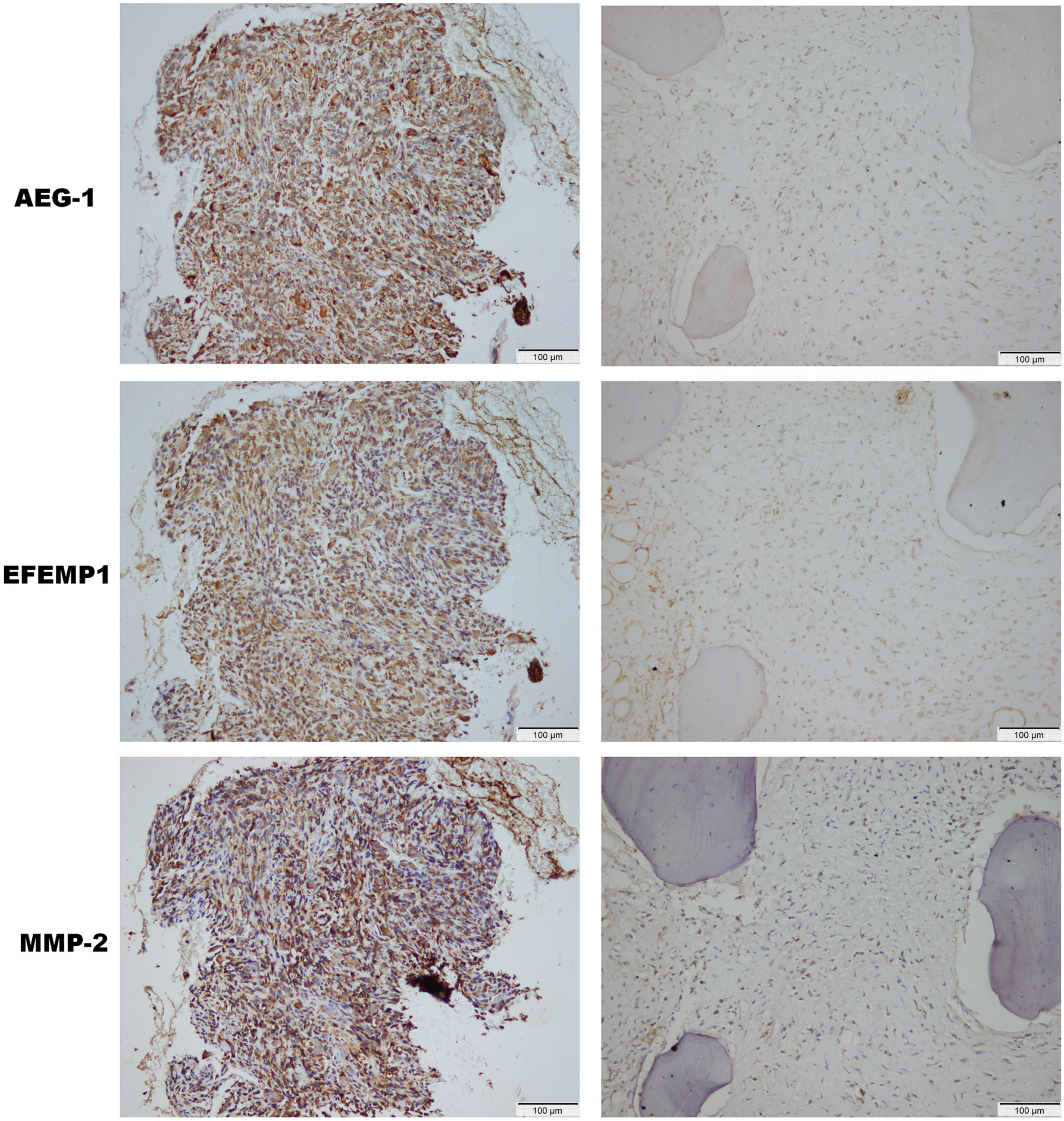
AEG-1
EFEMP1
MMP-2
100 μm

Figure S9: The relationship among EFEMP1, AEG-1 and MMP-2 in osteosarcoma patients. There was a strong correlation between EFEMP1, AEG-1 and MMP-2 in human osteosarcoma by immunohistochemical staining. (left) A case of high AEG-1 expression together with high EFEMP1 and MMP-2 expression. (right) A case of low AEG-1 expression together with low EFEMP1 and MMP-2 expression.

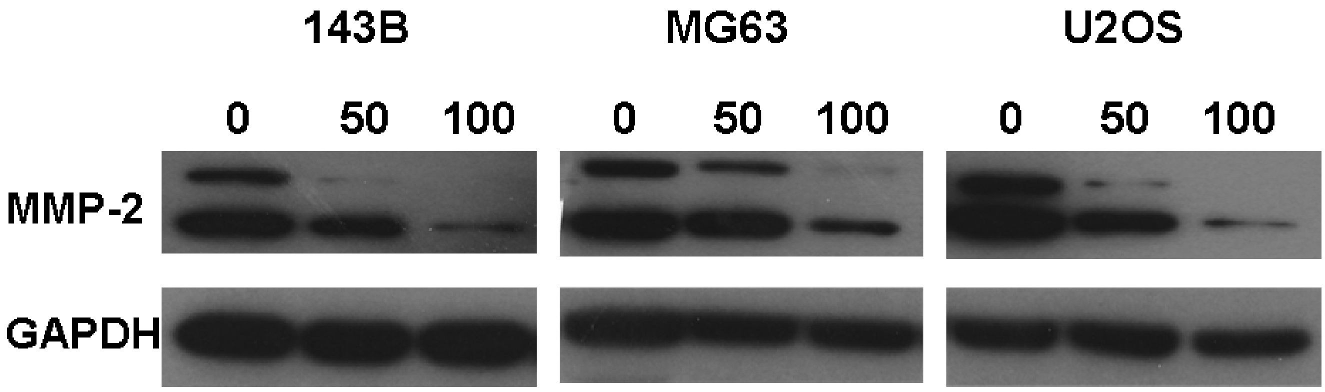

Figure S10: PDTC suppresses MMP-2 expression via inhibition of NF-κB signaling pathway in osteosarcoma cells. The basal level of MMP-2 was decreased by treatment with 50μM and 100μM specific NF-κB inhibitor-PDTC in 143B, MG63 and U2OS osteosarcoma cells, analyzed by western blot. Protein expression levels were normalized to GADPH.
